# Pharmacological reactivation of autophagic flux by natural compounds or synthetic cell-permeable peptide prevents doxorubicin-induced cardiomyopathy

**DOI:** 10.1007/s00395-026-01174-9

**Published:** 2026-03-29

**Authors:** Leonardo Schirone, Daniele Vecchio, Valentina Valenti, Vittorio Picchio, Sonia Schiavon, Luca D’Ambrosio, Flavio di Nonno, Selenia Miglietta, Michela Relucenti, Luca Madaro, Silvia Palmerio, Claudia Cozzolino, Margherita Litterio, Gianmarco Sarto, Beatrice Simeone, Nicola Moro, Shazia Tahir, Tania Zaglia, Giuseppe Biondi Zoccai, Elena De Falco, Vincenzo Petrozza, Ernesto Greco, Giacomo Frati, Maurizio Forte, Sebastiano Sciarretta

**Affiliations:** 1 Department of Health and Life Sciences, European University of Rome, Rome, Italy; 2 Department of Medical and Surgical Sciences and Biotechnologies, Sapienza University of Rome, Corso Della Repubblica 79, Latina, Italy; 3 Maria Cecilia Hospital, GVM Care and Research, Cotignola, Italy; 4 Present Address: Department of Angiocardioneurology and Translational Medicine, IRCCS Neuromed, Via Atinense 18, 86077 Pozzilli, Italy; 5 Department of Anatomical, Histological, Forensic Medicine and Orthopaedic Sciences, Sapienza University of Rome, Rome, Italy; 6 Department of Medicine, University of Verona School of Medicine, Verona University Hospital Trust, Verona, Italy; 7 Cardiology Division, ICOT Istituto “Marco Pasquali” University Hospital, Latina, Italy; 8 Department of Biomedical Sciences, University of Padua, Padua, Italy; 9 University of Palermo, Palermo, Italy

**Keywords:** Anthracyclines, Doxorubicin, Autophagy, Trehalose, Spermidine, Heart failure

## Abstract

Therapeutic strategies to limit doxorubicin (DOX)-induced cardiomyopathy are still limited due to incomplete characterization of the underlying molecular mechanisms. The exogenous activation of autophagy was reported to exert cardioprotective effects in preclinical models of cardiovascular diseases. We tested whether restoration of autophagy by different pharmacological approaches can reduce DOX-induced cardiomyopathy. A validated preclinical murine model of DOX-induced cardiotoxicity (final cumulative dose of 15 mg/kg) was used to test the cardiac effects of both natural (trehalose and spermidine) and synthetic (Tat-Beclin 1 D11) activators of autophagy. Cardiac function was evaluated by echocardiographic analyses. We performed histological analyses (Masson trichrome staining, TUNEL assay) to investigate fibrosis and apoptosis. Biomolecular analyses, confocal and transmission electron microscopy were used to assess levels of autophagy, autophagic flux, mitophagy and mitochondrial alterations. We also evaluated the effects of autophagy activators in a validated syngeneic model with subcutaneous injection of breast cancer cells treated with DOX. We found that DOX-induced cardiotoxicity is associated with impaired autophagic flux (accumulation of LC3-II and p62). Reactivation of autophagic flux by trehalose or spermidine rescues cardiac function and cardiomyocyte survival in mice treated with DOX, along with an overall amelioration of mitochondrial health and mitophagy. Selective reactivation of autophagy by Tat-Beclin 1 D11 also recapitulates the protective effects exerted by trehalose and spermidine. Autophagy activators preserve cardiac function without affecting the antineoplastic effects of DOX in mice with cancer. Boosting autophagic flux is a suitable therapeutic approach to prevent cardiotoxicity induced by anthracyclines.

**Graphical abstract:**

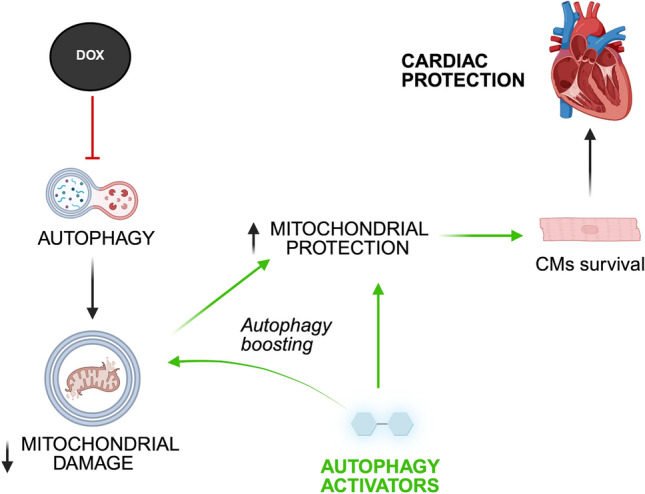

**Supplementary Information:**

The online version contains supplementary material available at 10.1007/s00395-026-01174-9.

## Introduction

Doxorubicin (DOX) is a highly potent antineoplastic agent from the anthracycline class. Despite its significant side effects, it remains widely used to treat various malignancies. The development of cardiomyopathy is a common side effect of DOX, affecting nearly 30% of patients within five years of receiving chemotherapy in a dose-dependent manner [39]. Although numerous studies were performed to understand the molecular mechanisms underlying myocardial toxicity induced by DOX, our understanding of this phenomenon remains inadequate to limit cardiac side effects [45].

Autophagy is an evolutionarily conserved cytoplasmic mechanism for the degradation and turnover of senescent or damaged proteins and organelles in eukaryotic cells. This homeostatic process involves the sequestration and lysosomal digestion of portions of the cytoplasm encapsulated in lipid-membrane vesicles known as autophagosomes [29]. Preclinical evidence demonstrated that inhibition of autophagy concurs with the development of several cardiovascular diseases (CVDs), whereas a physiological reactivation of this process was reported to reduce cardiac injury in response to stress [34, 36]. Of note, an impairment of autophagic flux was recently recognized as one of the potential mechanisms underlying DOX-induced cardiomyopathy [25]. Therefore, pharmacological restoration of autophagy could represent a valuable translational approach to enhance cardiovascular health in cancer patients.

Among pharmacological activators, caloric restriction mimetics have emerged as highly effective autophagy inducers with extraordinary anti-aging effects and therapeutic effects in multiple organ diseases [12, 33]. Previous work demonstrated that trehalose (TRE), a natural disaccharide, attenuates neurological, hepatic, renal, and cardiovascular diseases [6, 30]. We previously found that TRE reduces post-ischemic cardiac remodeling through the activation of autophagy [35]. Spermidine (SP), a polyamine derived from putrescine and initially isolated from semen, also proved to exert exceptional beneficial effects against aging, significantly extending mammalian lifespan, and against cardiovascular stress, reducing cardiac senescence, pressure overload-induced heart failure and atherosclerosis in preclinical models of CVD, through autophagy activation [7, 8]. Importantly, TRE and SP are FDA-approved dietary supplements with negligible side effects.

In this study, we investigated, for the first time, the effects of TRE and SP administration on cardiac function and mitochondrial damage in a clinically relevant murine model of DOX-induced cardiomyopathy. We also tested for the first time the effects of the synthetic peptide Tat-Beclin 1 D11, a highly specific stimulator of autophagy directly targeting the autophagy machinery, to specifically test the effects of autophagy activation. Finally, we tested whether trehalose, spermidine, and Tat-Beclin 1 D11 interfere with the antitumor activity of DOX in a cancer mouse model.

## Materials and methods

All experimental procedures are detailed in Supplementary Material.

### Animal models and ethics

8–12-week-old C57BL/6 J, C57BL/6N wild-type mice and α-MHC-MitoTimer ± (C57BL/6-Tg(Myh6-DsRed1*/COX8A)40830Rag/J – JAX stock no. 028715) mice were acquired from The Jackson Laboratory and Charles River. Both male and female mice were used in the experiments. These animals were bred and housed in similar conditions, in compliance with EU regulations and in adherence to the principles of the 3Rs. All experimental groups were housed in the same conditions: same sex siblings were housed together, up to four mice per cage. The investigation conformed to the Guide for the Care and Use of Laboratory Animals published by the US National Institutes of Health (NIH Publication No. 85–23, revised 1985). All procedures were approved by the Istituto Superiore di Sanità (Authorization number 147/2022-PR; 324/2023-PR).

### In vivo treatment, echocardiography and tissue harvesting

Heterozygous MitoTimer or C57BL/6 J mice (WT) were administered DOX (Sigma) intraperitoneally (i.p.) 5 mg/kg on days 0, 7, and 14, reaching a final cumulative dose of 15 mg/kg. For six weeks, mice received either water, water supplemented with 2% TRE (Sigma), 2% sucrose (Sigma), or 3 mM spermidine (Sigma). In addition, TRE treatment included the i.p. injection of 10% TRE at 1 g/kg dissolved in physiological saline, three times a week for the entire treatment duration. In a separate set of experiments, mice were treated with Tat-Beclin 1 D11 (Novus Biologicals) (three weekly i.p. injections at a dose of 15 mg/kg) (Supplementary Tables 1–3). Six weeks after the first injection of DOX, mice were subjected to echocardiographic assessment of cardiac structure (measuring the anterior wall, posterior wall, and septum) and function (fractional shortening, ejection fraction, heart rate), as previously described [32], using the VEVO3100 (Fujifilm Visualsonic) and its dedicated software. In a subset of experiments, we also evaluated global longitudinal strain (GLS), a parameter which is less affected by afterload changes. Subsequently, mice were euthanized, and hearts were collected for post-mortem analysis, which included the evaluation of autophagy and autophagic flux, fibrosis, apoptosis, mitochondrial damage, mitophagy, and mitochondrial biogenesis.

### Breast cancer model

A syngeneic model with subcutaneous injection of breast cancer cells was developed as previously described in literature [22, 23, 25]. The murine EO771 breast carcinoma cell line (ATCC, CRL-3461) was cultured in complete growth medium under standard conditions (37 °C, 5% CO₂). For tumour implantation, cells were harvested during the logarithmic growth phase, washed twice in sterile phosphate-buffered saline (PBS), and resuspended in PBS at the appropriate concentration. 1 × 10^5^ EO771 cells in 100 µL PBS were injected subcutaneously into the right flank of each mouse using a 27G needle. Tumour growth was monitored starting on day 7 post-implantation. At day 7, tumour size was measured in all animals using digital calipers, and tumour volume was calculated as described below. Mice were then stratified based on tumour volume and allocated to experimental groups to ensure comparable mean baseline tumour burden across groups. Group allocation was performed to minimize intergroup variability in tumour size at treatment initiation (day 7). The following groups were generated: untreated control (CTR NT), doxorubicin alone (DOX), DOX + Trehalose (TRE), DOX + Tat-Beclin 1 D11 (TAT), and DOX + spermidine (SP). Each experimental group included 12–20 mice. Tumour dimensions were measured at regular intervals using digital calipers until day 28 post-implantation. Tumour volume was calculated using the formula: Tumor volume = (length × width^2^)/2. Length represents the longest tumour diameter, and width represents the perpendicular diameter. Tumour volume was expressed in mm^3^. DOX-untreated mice were sacrificed after four weeks for ethical reasons. Cardiac function was evaluated as previously described at weeks 4 and 6 after the first DOX injection. Tumours were harvested at the experimental endpoint. Excised tumours were fixed in 4% paraformaldehyde, paraffin-embedded, and sectioned at 10 µm thickness. Paraffin sections were deparaffinized, rehydrated, and stained with hematoxylin and eosin (H&E) according to standard protocols. Slides were dehydrated, mounted, and imaged using a brightfield microscope.

### Statistical analyses

All data are presented as mean ± standard error of the mean (SEM). A two-sided Student’s t-test was used to compare two independent groups involving continuous variables. When comparing more than two independent groups, a one-way ANOVA followed by a post-hoc test was employed. Tumour growth curves were analysed using two-way ANOVA with repeated measures, followed by Bonferroni post-hoc multiple comparison test. All statistical analyses were performed using GraphPad Prism software (GraphPad Software, San Diego, CA, USA), and a *p*-value < 0.05 was considered statistically significant.

## Results

### Trehalose protects the heart from DOX cardiotoxicity and reactivates autophagic flux.

**Fig. 1:**
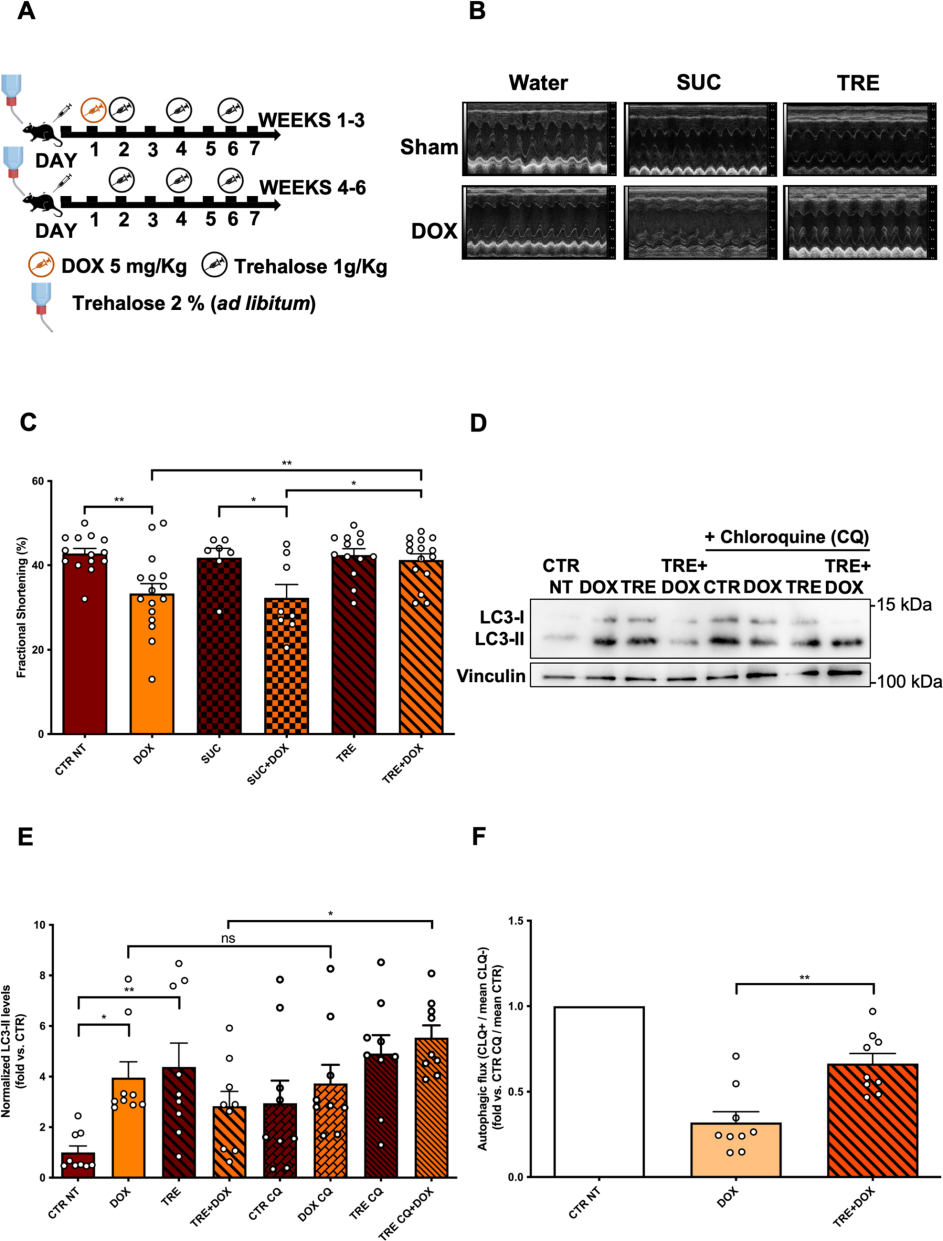
Trehalose protects the heart from DOX cardiotoxicity by activating autophagy. **A** Wild-type mice were treated for 6 weeks with doxorubicin (DOX) 15 mg/kg and sucrose/trehalose (2% in drinking water). **B**, **C** M-mode echocardiographic analyses of myocardial fractional shortening. Data represent mean ± SEM (N = 7–16); **D**–**F** Evaluation of autophagy and autophagic flux. Representative western blot for the autophagy marker LC3-II (**D**) in myocardial lysates and corresponding quantification (**E**). A subgroup of mice received chloroquine 4 h before sacrifice to assess the autophagic flux (CLQ + group/CLQ- matching group mean normalized LC3-II expression ratio). Data represent mean ± SEM (N = 9). In panel **F** the CTR NT group was arbitrarily set to 1 as a reference. Data were analysed with one-way ANOVA with a Bonferroni correction (**C**, **E**) or a two-tailed Student’s t-test (**F**). *P ≤ 0.05; **P ≤ 0.01; ns = non-significant (*P* > 0.05). Legend: CTR NT = Control no-treatment mice; DOX = Control mice treated with DOX; TRE = mice treated with trehalose; TRE + DOX = mice treated with DOX and trehalose; SUC = mice treated with sucrose; SUC + DOX = mice treated with sucrose and DOX; TRE CQ = mice treated with trehalose and CLQ; TRE CQ + DOX = mice treated with DOX, trehalose and CLQ; CTR CQ = Control mice treated with CLQ; DOX CQ = Control mice treated with DOX and CLQ

We studied the effects of TRE administration in a preclinical and validated murine model of DOX-induced cardiotoxicity (15 mg/kg of DOX administered as three intraperitoneal injections of 5 mg/kg once a week) [25, 32] (Fig. 1A). TRE administration was compared to water (no treatment) or sucrose treatment (control disaccharide). Six weeks after the initial DOX injection, a significant decline of systolic function was observed in mice receiving water or sucrose, as evidenced by decreased fractional shortening, ejection fraction, and global longitudinal strain (GLS) (Fig. 1B, C; Supplementary Fig. 1 A, B). In contrast, mice receiving TRE exhibit a preserved systolic function following DOX treatment (Fig. 1B, C; Supplementary Fig. 1A, B). These data demonstrate that TRE prevents DOX-induced cardiotoxicity.

Hearts were harvested from mice, and we found a reduction in heart mass in mice that received DOX. However, DOX-treated mice receiving TRE do not show changes in heart mass (Supplementary Fig. 1C). We did not observe differences in heart rate among all experimental groups (Supplementary Fig. 1D). Protein lysates were then prepared to evaluate autophagy and autophagic flux. TRE increases baseline levels of LC3-II, an established marker of autophagosome abundance, compared with controls. Interestingly, we also observed that DOX treatment results in a marked accumulation of LC3-II, which tends to be attenuated by TRE (Fig. 1D, E). LC3-II accumulation may be secondary to either increased autophagosome formation or decreased autophagosome-lysosome fusion, namely a reduced autophagic flux. To investigate the autophagic flux, mice were treated with the autophagic flux inhibitor chloroquine (CQ), administered four hours before sacrifice, as indicated by Guidelines for Autophagy Evaluation [20]. We observed that LC3-II does not significantly increase in mice receiving DOX + CQ as compared to mice receiving DOX (Fig. 1D, E). In contrast, DOX-treated mice receiving TRE and CQ show the highest levels of LC3-II, with a significantly higher amount than the TRE group without CQ. Remarkably, direct measurement of autophagic flux confirmed that DOX treatment markedly inhibits autophagic flux, which is completely rescued by TRE treatment (Fig. 1F). These results were also corroborated by the evaluation of protein levels of p62 (SQSTM1), which is degraded when autophagy flux is increased, in the heart of mice with and without DOX treatment. Levels of p62 increase in the heart of DOX-treated mice, confirming autophagy inhibition, but this accumulation is attenuated in mice treated with DOX and TRE (Supplementary Fig. 2A, B).

To investigate whether TRE restores autophagic flux in DOX-treated cardiomyocytes using an independent validated method, we performed additional in vitro experiments in primary cardiomyocytes with adenovirus-mediated mRFP-GFP-LC3 overexpression, again in keeping with Guidelines for Autophagy Assessment [20]. The mRFP-GFP-LC3 construct allows visualization of autophagosomes (green and red dots, yellow when overlaid) and autophagosomes fused with lysosomes, namely autophagolysosomes (red-only dots, since GFP is sensitive to low pH and proteases and its signal is quenched in lysosomes). A reduction in autophagolysosome number indicates inhibition of autophagic flux [19]. We found that DOX treatment significantly reduces the number of both red (autophagolysosomes) and yellow (autophagosomes) dots, whereas co-treatment with TRE significantly restores them, particularly autophagolysosome number (Supplementary Fig. 3A, B). These data confirm that DOX inhibits cardiomyocyte autophagic flux, which is restored by TRE.

We also found an increased LC3-II level in cardiomyocytes treated with DOX or TRE in vitro, compared with control cells (Supplementary Fig. 3C, D). However, LC3-II level does not increase in cardiomyocytes treated with DOX plus bafilomycin (i.e. lysosome inhibitor), as compared to DOX treatment alone. Conversely, bafilomycin significantly increases LC3-II levels in control cardiomyocytes not treated with DOX or in cardiomyocytes treated with TRE compared with cardiomyocytes exposed to the same conditions without bafilomycin co-treatment. These data confirm that DOX impairs autophagy flux in cardiomyocytes, whereas TRE stimulates it (Supplementary Fig. 3C, D). To further corroborate our findings, we assessed the activity of TFEB, a transcriptional regulator of autophagy that is activated by TRE in the heart [35]. When activated, TFEB translocates into the nucleus, where it activates genes involved in autophagy promotion and lysosomal biogenesis. We observed that DOX reduces TFEB nuclear accumulation, whereas this effect is not observed in mice receiving DOX plus TRE (Supplementary Fig. 4).

### Trehalose prevents DOX-induced myocardial damage.

**Fig. 2:**
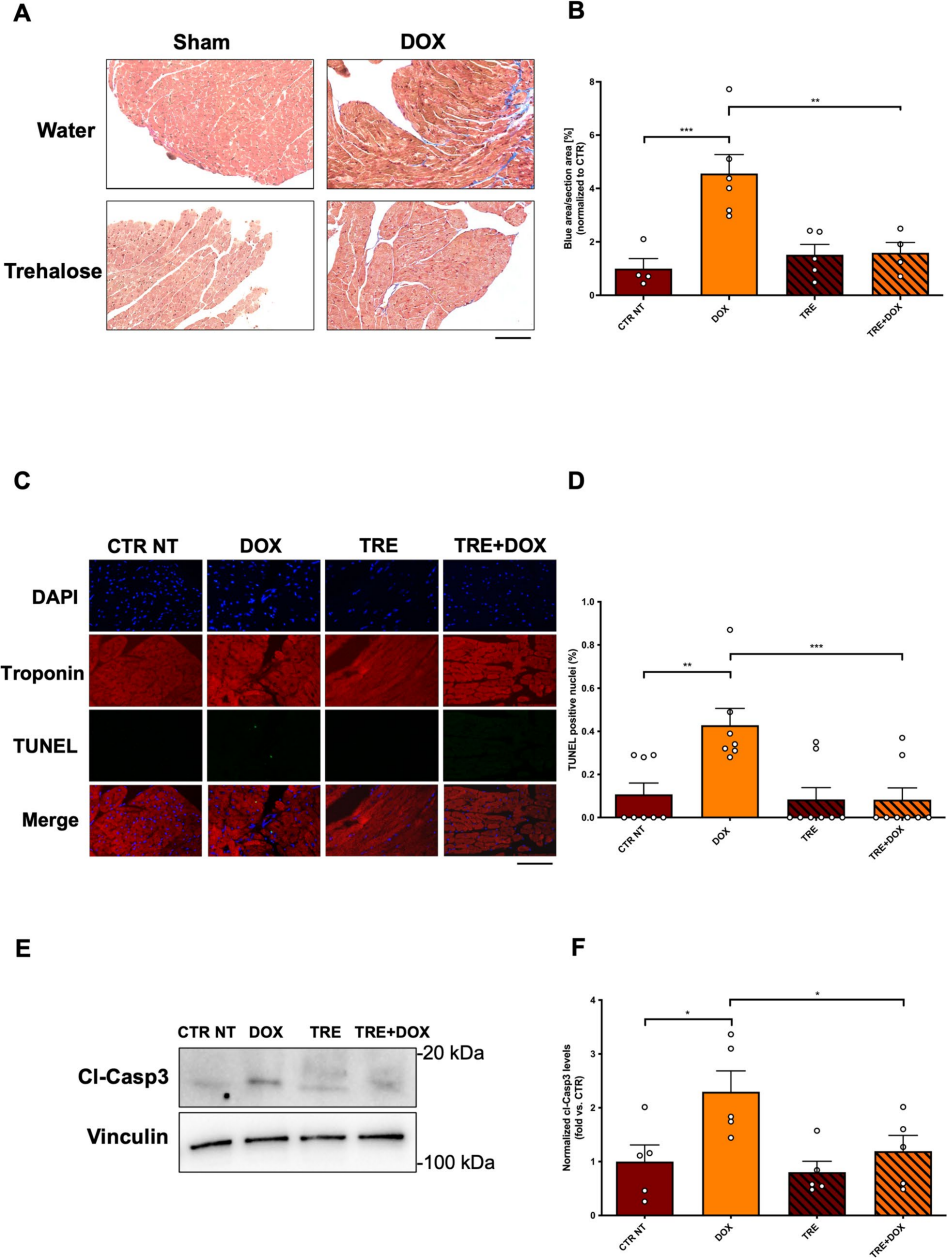
Trehalose prevents DOX-induced myocardial damage. **A**, **B** Heart specimens were sliced into 5 µm thick sections and stained with Masson’s trichrome staining. Collagen deposition was calculated by quantifying the ratio of blue-stained area to total section area. Data represent mean ± SEM (N = 4–6). Scalebar = 200 µm; **C**, **D** Cell death was evaluated in myocardial sections by quantifying the percentage of TUNEL-positive nuclei. Only troponin-positive cells were included in this evaluation. Data represent mean ± SEM (N = 7–8). Scalebar = 100 µm; **E**, **F** Representative western blot for cleaved caspase 3 (**E**) in myocardial protein lysates and corresponding quantification (**F**). Data represent mean ± SEM (N = 5). Data were analysed with one-way ANOVA with a Bonferroni post-hoc test. *P ≤ 0.05; **P ≤ 0.01; ***P ≤ 0.001. Legend: CTR NT = Control no-treatment mice; DOX = Control mice treated with DOX; TRE = mice treated with trehalose; TRE + DOX = mice treated with DOX and trehalose

To better understand the effects of autophagy stimulation in the myocardium by TRE, we also evaluated structural cardiac changes following DOX treatment. While control mice show initial signs of interstitial fibrosis after DOX administration, those treated with TRE display no evidence of parenchymal alterations or collagen deposition (Fig. 2A, B). To verify whether fibrosis is driven by cardiomyocyte cell death, we evaluated levels of cardiac apoptosis by TUNEL assay, revealing an increase in TUNEL-positive nuclei in the hearts of DOX-treated mice, compared with mice receiving DOX + TRE (Fig. 2C, D). Levels of the apoptotic marker cleaved caspase 3 also increase in the heart of mice treated with DOX, but TRE treatment blunts cleaved caspase 3 accumulation (Fig. 2E, F).

These data suggest that TRE abrogates DOX-induced myocardial abnormalities by reducing cardiac apoptosis and fibrosis.

### Trehalose administration induces mitophagy and clears damaged mitochondria.

**Fig. 3:**
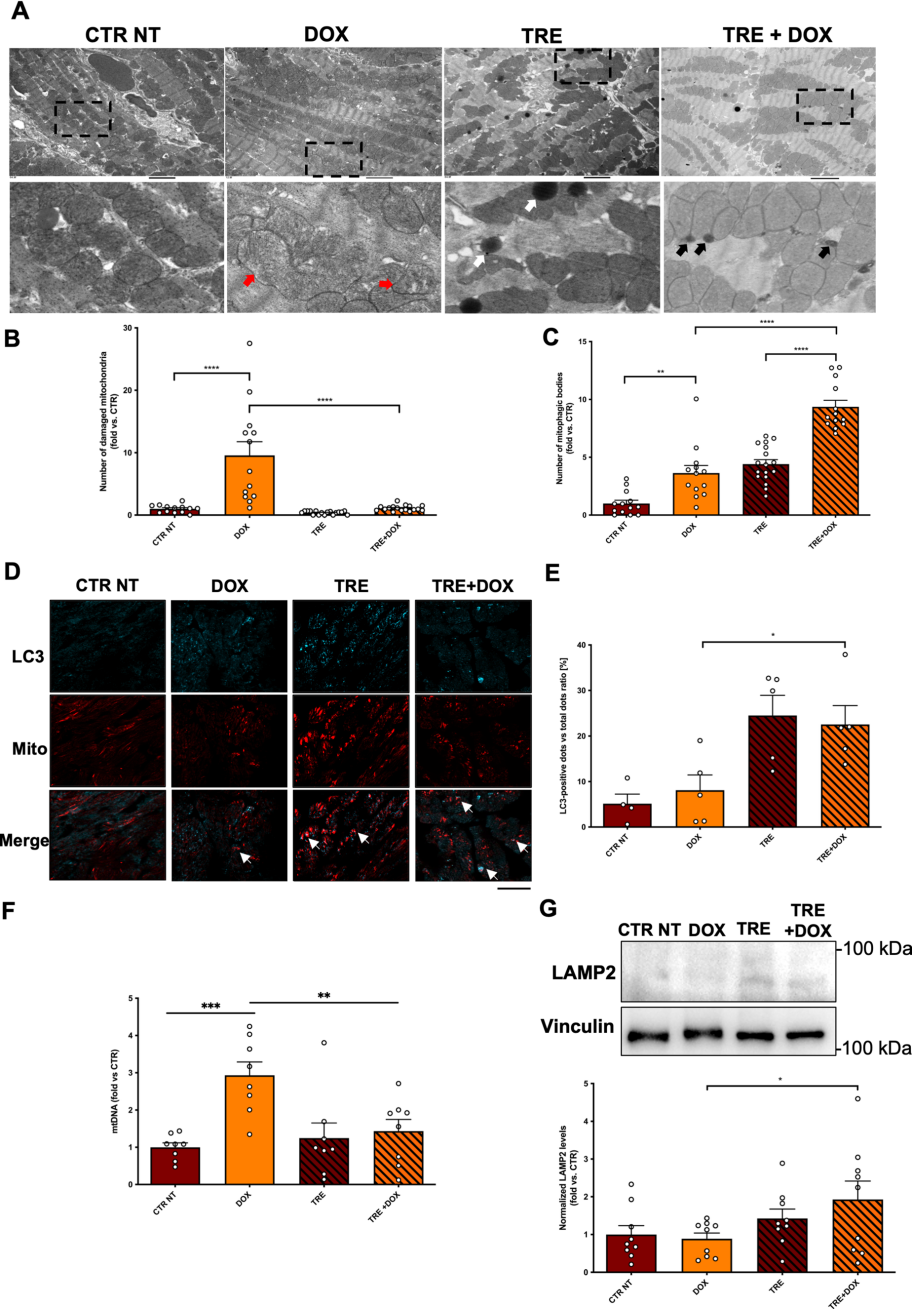
Trehalose administration induces mitophagy and clears damaged mitochondria. **A** Representative TEM images of myocardial mitochondria from mice treated with DOX and trehalose 6 weeks after the first administration. Higher magnification fields are provided (bottom row). Scalebar: 2 μm. White arrows point to lysosomes, black arrows point to lysosomes fusing with mitochondria or late mitophagic bodies, red arrows point to damaged mitochondria; **B** quantification through TEM imaging of number of damaged mitochondria and **C** mitophagic bodies percentage out of total counted mitochondria (mitochondrial damage: N = 13–17 microscopic fields from 3 independent samples; mitophagic bodies: N = 13–20 microscopic fields from 3 independent samples); **D**, **E** Confocal microscopy of MitoTimer mouse myocardium stained with an LC3-II antibody. Co-localization of spontaneous mitochondrial fluorescence and LC3-II autophagosome marker was quantified (**E**). White arrows point to co-localized mitochondrial and autophagic signals. Scalebar = 50 µm (N = 4-5). **F** Relative mitochondrial DNA (mtDNA) content in mouse hearts, evaluated by polymerase chain reaction for cytochrome b (N = 8). **G** Representative western blot for LAMP-2 and corresponding quantification; (N = 9); Data represent mean ± SEM. Data were analysed with one-way ANOVA with a Bonferroni post-hoc test. *P ≤ 0.05; **P ≤ 0.01; ***P ≤ 0.001; ****P ≤ 0.0001. Legend: CTR NT = Control no-treatment mice; DOX = Control mice treated with DOX; TRE = mice treated with trehalose; TRE + DOX = mice treated with DOX and trehalose

Transmission electron microscopy (TEM) analyses were conducted to investigate potential morphological changes in mitochondria within cardiomyocytes. These analyses reveal severe mitochondrial abnormalities, such as alterations in mitochondrial cristae density and morphology, in the myocardium of DOX-treated mice, whereas these abnormalities are not detected in either control or TRE-treated groups (Fig. 3A, B). An increased prevalence of mitochondria encapsulated within autophagosomal vesicles is also noted across all DOX-treated groups. Remarkably, mice treated with TRE exhibit a significantly higher number of free lysosomes and autophagic bodies containing mitochondrial fragments, with the most pronounced increase found in TRE-treated mice that had also received DOX (Fig. 3C).

To further corroborate this evidence, myocardial sections from DOX-treated MitoTimer mice, which exhibit innate mitochondrial fluorescence (as described in the Methods section) [40], were stained with an LC3 antibody. The analysis indicated that TRE treatment enhances the colocalization of mitochondrial and LC3 signals, implying a stimulation of mitophagy, the selective form of autophagy for the removal of dysfunctional mitochondria (Fig. 3D, E). The impairment of mitophagy by DOX was further corroborated by the observation of increased mitochondrial mass in the heart, as assessed by the evaluation of mtDNA. Notably, TRE rescue mtDNA levels in mice receiving DOX (Fig. 3F**)**.

Finally, we found that protein levels of LAMP2, a marker of lysosomal abundance and function, are increased in the hearts of mice receiving TRE compared with mice without TRE treatment (Fig. 3G)**,** suggesting that the stimulation of autophagic flux by TRE may also be supported by increased lysosomal biogenesis/function.

Together, these data suggest that TRE protects the myocardium by facilitating the removal of damaged mitochondria through enhanced mitophagy.

### Trehalose reduces DOX-induced mitochondrial biogenesis.

Subsequent investigations focused on mitochondrial biogenesis in MitoTimer mice, a model wherein mitochondria emit green fluorescence within the first 48 h post-biogenesis before transitioning to red [13]. Using this model, we observed an increase in mitochondrial biogenesis in the myocardium of DOX-treated mice that did not receive TRE, a phenomenon that was not seen in the myocardium of mice treated with TRE and DOX combination (Supplementary Fig. 5A, B). These observations were further supported by the detection in the heart of DOX-treated mice of increased protein levels of peroxisome proliferator-activated receptor gamma coactivator 1-alpha (PGC-1α), which is recognized as a pivotal regulator of mitochondrial biogenesis (Supplementary Fig. 5C, D) [2]. Mice receiving DOX and TRE do not show increased levels of PGC-1α.

These findings suggest that cardiomyocytes may significantly upregulate mitochondrial biogenesis as a compensatory mechanism to replace mitochondria that are damaged by DOX. However, the induction of autophagic flux and mitophagy appears to mitigate this response, highlighting the complex interplay between mitochondrial biogenesis and autophagy in the context of DOX-induced cardiac stress.

### Spermidine protects the heart from DOX-induced cardiotoxicity and reactivates autophagy.

To corroborate the trehalose findings, we tested whether SP, a different caloric restriction mimetic and autophagic flux inducer, protects the heart from DOX-induced toxicity. Of note, TRE and SP were previously found to activate autophagic flux through different molecular mechanisms. TRE activates the transcription factor TFEB, a critical promoter of autophagosomal and lysosomal biogenesis, whereas SP inhibits p300, an acetyltransferase and potent autophagy inhibitor [27, 37].

**Fig. 4:**
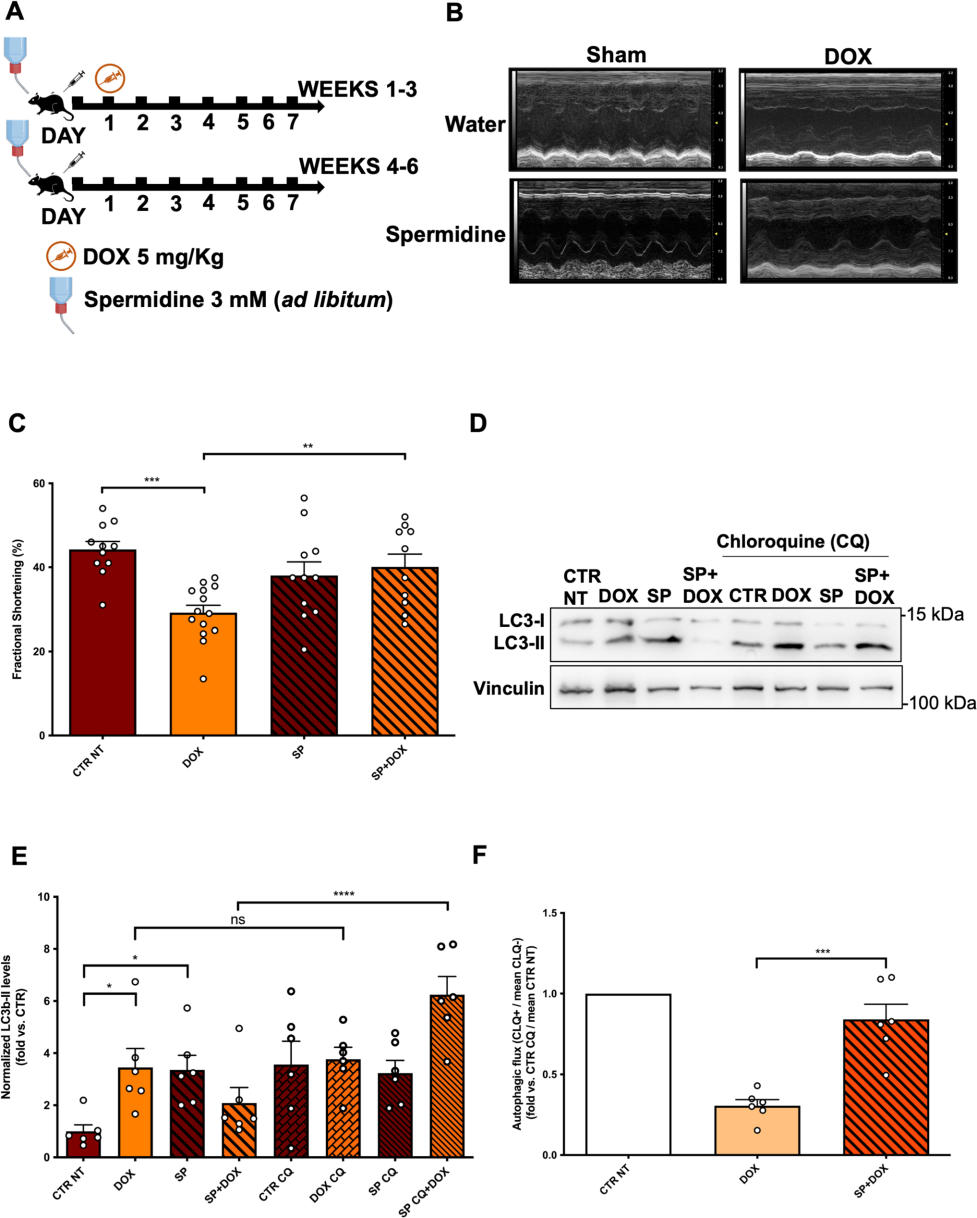
Spermidine protects the heart from DOX cardiotoxicity by activating autophagy. **A** Wild-type mice were treated for 6 weeks with doxorubicin (DOX) 15 mg/kg and spermidine (3 mM in drinking water). **B**, **C** M-mode echocardiographic analyses of myocardial fractional shortening. Data represent mean ± SEM (N = 10–14); **D**–**F** Evaluation of autophagy and autophagic flux. Representative western blot for the autophagy marker LC3-II (**D**) in myocardial lysates and corresponding quantification (**E**). A subgroup of mice received chloroquine 4 h before sacrifice to assess the autophagic flux (CLQ + groups/CLQ-matching group mean normalized LC3-II expression ratio) (**F**) (N = 6). In panel **F** the CTR NT group was arbitrarily set to 1 as a reference. Data represent mean ± SEM. Data were analysed with one-way ANOVA with a Bonferroni correction (**C, E**) or a two-tailed Student’s t-test (**F**). *P ≤ 0.05; **P ≤ 0.01; ***P ≤ 0.001; ****P ≤ 0.0001; ns = non-significant (*P* > 0.05). Legend: CTR NT = Control no-treatment mice; DOX = Control mice treated with DOX; SP = mice treated with spermidine; SP + DOX = mice treated with spermidine and DOX; SP CQ = mice treated with spermidine and CLQ; SP CQ + DOX = mice treated with spermidine, DOX and CLQ; CTR CQ = Control mice treated with CLQ; DOX CQ = Control mice treated with DOX and CLQ

We found that SP treatment (Fig. 4A) attenuates cardiac dysfunction in mice treated with DOX (Fig. 4B, C) (Supplementary Fig. 1A, B). We then assessed the effects on autophagic flux and observed that SP also recovers autophagic flux in the heart of DOX-treated mice. In fact, SP significantly increases LC3-II levels at baseline and in DOX-treated mice receiving CQ, as compared to those not receiving CQ (Fig. 4D, E). In contrast, DOX induces cardiac LC3-II accumulation, which is not further increased in response to CQ treatment. Autophagic flux measurement confirms that DOX treatment reduces cardiac autophagic flux, which is significantly restored by SP co-treatment (Fig. 4F). We also evaluated the effects of SP on p62 levels in CTR and DOX-treated mice and found that it attenuates DOX-induced cardiac accumulation of p62 (Supplementary Fig. 2C, D), further indicating that SP restores autophagic flux in the heart of DOX-treated mice. We also assessed EP300 activity, a well-known acetyltransferase that negatively regulates autophagy. Previous studies showed that SP reduces EP300 activity without affecting its expression, thereby activating autophagy [27]. Consistently, we found that the protein acetylation status is decreased in the hearts of mice treated with spermidine, either with or without DOX (Supplementary Fig. 6).

### Tat-Beclin 1 D11, a specific autophagy-activating peptide, protects the heart from DOX cardiotoxicity

**Fig. 5:**
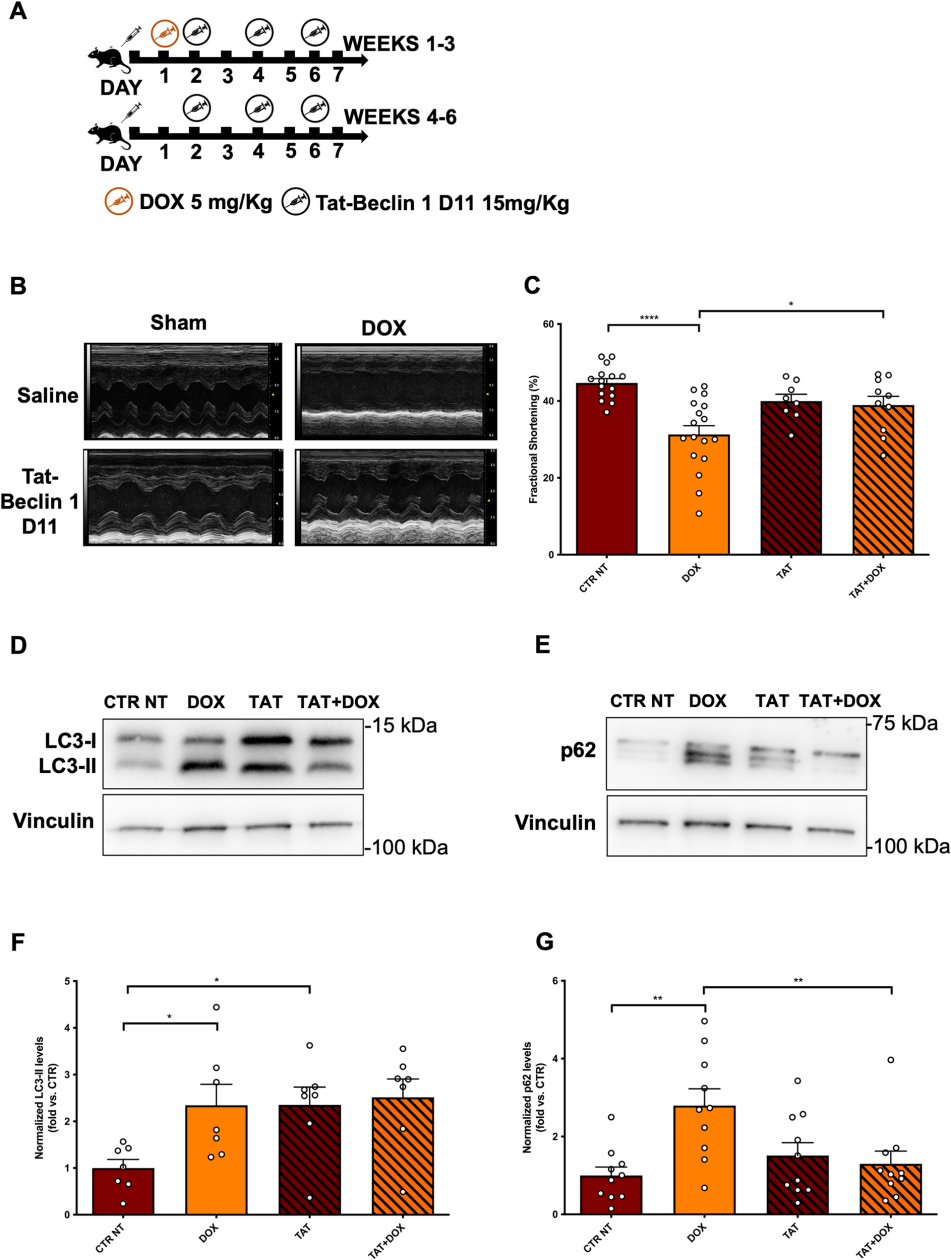
Tat-Beclin 1 D11 protects the heart from DOX cardiotoxicity by activating autophagy. **A** Wild-type mice were treated for 6 weeks with doxorubicin (DOX) 15 mg/kg and Tat-Beclin 1 D11 (15 mg/kg). **B**, **C** M-mode echocardiographic analyses of myocardial fractional shortening. Data represent mean ± SEM (N = 8–17); **D**–**G** Evaluation of autophagy. Representative western blots for the autophagy markers LC3-II and p62 (**D, E**) in myocardial lysates and corresponding quantification (**F, G**). Data represent mean ± SEM (LC3: N = 7; p62: N = 10). Data were analysed with one-way ANOVA with a Bonferroni post-hoc test. *P ≤ 0.05; **P ≤ 0.01; ****P ≤ 0.0001. Legend: CTR NT = Control no-treatment mice; DOX = Control mice treated with DOX; TAT = mice treated with Tat-Beclin 1 D11; TAT + DOX = mice treated with DOX and Tat-Beclin 1 D11

Finally, we wanted to test whether a selective reactivation of autophagy could also rescue DOX-induced cardiotoxicity. We treated mice with the synthetic autophagy-stimulating peptide Tat-Beclin 1 D11 (Fig. 5A), a highly specific inducer of autophagic flux that directly interferes with the autophagy machinery by targeting Beclin-1 protein, without non-specific autophagy-independent functions. We observed that Tat-Beclin 1 D11 also preserves cardiac function in mice treated with DOX (Fig. 5B, C). Biomolecular analyses on protein lysates from heart lysates also revealed that Tat-Beclin 1 D11 administration activates autophagy in the heart and reduces cardiac p62 levels in DOX-treated mice (Fig. 5D–G), indicating a reactivation of autophagic flux.

### Autophagy activation does not affect the antineoplastic effects of DOX

**Fig. 6:**
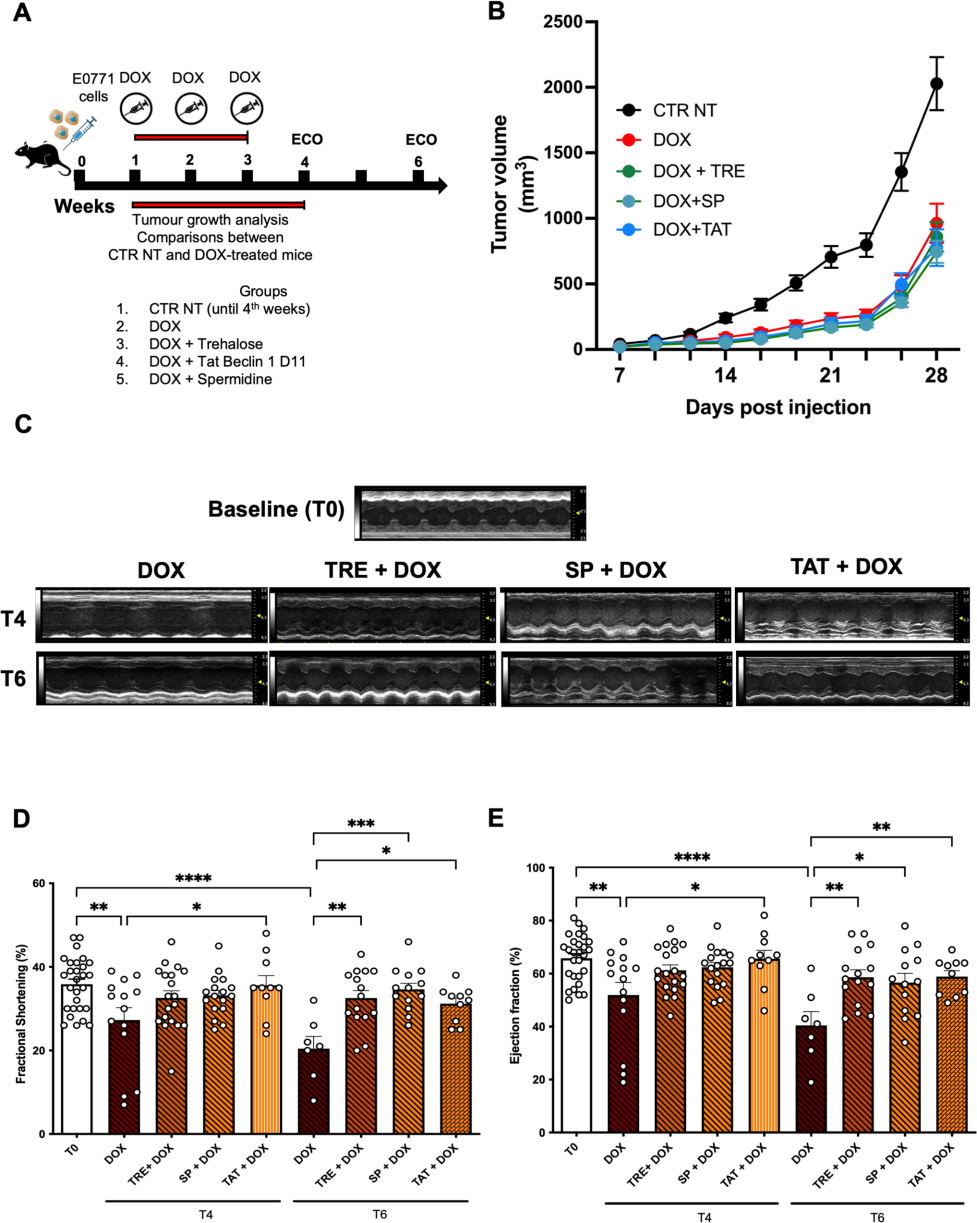
Autophagy activators reduce DOX-induced cardiotoxicity without affecting its antineoplastic effects. **A** 1 × 10^5^ EO771 breast cancer cells were subcutaneously injected into wild-type (WT) C57BL/6N mice and then treated with a cumulative dose of doxorubicin (DOX, 15 mg/kg) administered as three weekly injections (5 mg/kg on days 7, 14, and 21 post-injection). Experimental groups included: untreated control (CTR NT), DOX, DOX + trehalose (TRE), DOX + spermidine (SP) and DOX + Tat-Beclin 1 D11 (TAT). Tumour volume (mm^3^) was measured at the indicated time points up to day 28 post-injection and echocardiographic analyses were performed after four and six weeks. **B** Tumour growth kinetics. Data are presented as tumor volume (mm^3^) over time. Each group included 12–20 mice. Data were analysed with two-way ANOVA with a Bonferroni post-hoc test. ***P ≤ 0.001 DOX versus CTR NT, DOX + TRE versus CTR NT, DOX + TAT versus CTR NT; ****P ≤ 0.0001 DOX + SP versus CTR NT at 28 days (**C**) Representative images of M-mode echocardiographic analyses and quantification of myocardial fractional shortening (**D**) and ejection fraction (**E**) at baseline (T0) and at 4 (T4) and 6 (T6) weeks after DOX treatment in mice with subcutaneous injection of E0771 breast cancer cells. Data represent mean ± SEM. (N = 28 at T0; T4: DOX N = 14, TRE + DOX N = 19, SP + DOX N = 17, TAT + DOX N = 10; T6: DOX N = 7, TRE + DOX N = 15, SP + DOX N = 13, TAT + DOX N = 10). Data were analysed with one-way ANOVA with a Bonferroni post-hoc test. *P ≤ 0.05; **P ≤ 0.01; ***P ≤ 0.001; ****P ≤ 0.0001; ns—not significant T0 versus TRE + DOX, T0 versus SP + DOX, T0 versus TAT + DOX at T4 and T6

Finally, we tested whether the activation of autophagy interferes with the antitumoral activity of DOX, while reducing its cardiotoxicity. To this end, we developed a model of subcutaneous injection of breast cancer cells and assessed tumour growth and cardiac function after DOX treatment, in the presence or absence of autophagy activators (Fig. 6A). Tumours were histologically confirmed (Supplementary Fig. 7). We observed that tumour volume was significantly decreased in mice treated with DOX with respect to untreated mice after 4 weeks of treatment. Remarkably, TRE, SP, and TAT did not affect the antineoplastic effect of DOX (Fig. 6B). On the other hand, echocardiographic analysis confirmed that autophagy reactivation preserves systolic function and reduces DOX-induced cardiotoxicity (Fig. 6C–E) (Supplementary Figs. 8 and 9).

## Discussion

The induction of cardiomyopathy by DOX remains a significant clinical challenge, underscoring the urgent need for effective cardioprotective strategies [43, 44]. The pathophysiology behind DOX-induced cardiotoxicity involves the generation of reactive oxygen species (ROS) leading to oxidative stress and mitochondrial dysfunction [15]. However, the molecular mechanisms underlying cellular and mitochondrial derangements induced by anthracyclines are complex and remain the subject of intense study [31, 47]. Our findings underscore the potential role of autophagic flux reactivation in mitigating DOX-induced cardiotoxicity in a clinically relevant murine model. TRE, a naturally occurring disaccharide that activates autophagy, has been studied for its remarkable bioprotective properties, including the stabilization of proteins and cellular membranes under stress conditions and was reported to improve cardiac remodeling after myocardial infarction in mice through autophagy activation [35]. Similarly, SP, a polyamine involved in cellular growth and proliferation, was shown to exert cardioprotective effects that primarily rely on autophagy stimulation [8, 12]. Our data significantly extend this evidence by demonstrating for the first time that both TRE and SP significantly preserve cardiac function in DOX-treated mice. Interestingly, the same effect was induced by the Tat-Beclin 1 D11 peptide, a highly selective activator of autophagic flux, which was previously shown to reduce I/R injury, pressure overload-induced cardiac remodeling, diabetic cardiomyopathy and stroke in preclinical animal models [9, 11, 38, 42]. Tat-Beclin 1 D11 is a synthetic autophagy-inducing peptide first developed in Levine’s laboratory, which stimulates autophagic flux by directly interfering with the autophagy machinery, without exerting autophagy-independent effects [38]. These data strongly support the notion that autophagic flux reactivation is the mechanism through which trehalose and spermidine prevent DOX-induced cardiomyopathy.

Our study fully aligns with previous evidence demonstrating that DOX impairs autophagic flux [24, 25]. Previous seminal work from Hill’s and colleagues first demonstrated that DOX blocks autophagic flux by reducing lysosome acidification [24]. Subsequent work from the Ghigo and Hirsch laboratories demonstrated that maladaptive phosphoinositide 3-kinases (PI3K)-γ signaling activation, which is triggered by toll-like receptor (TLR)-9 stimulation, is involved in autophagic flux block caused by DOX treatment [25]. Mechanistically, DOX causes the release of mitochondrial DNA fragments from damaged mitochondria, which act as damage-associated molecular patterns (DAMPs) and, in turn, activate TLR9. We also recently found that DOX treatment induces mitochondrial dysfunction and cardiotoxicity through the activation of Mammalian Sterile 20-like kinase (MST1) signaling, which also contributes to autophagic flux inhibition [32]. Aside from the molecular mechanisms promoting autophagy inhibition, our study demonstrates that a pharmacological reactivation of autophagic flux by different approaches consistently prevents cardiomyopathy induced by DOX. In this regard, TRE and SP appear to stimulate flux through different mechanisms. TRE is a major inducer of TFEB, a transcription factor promoting lysosomal biogenesis, whereas spermidine inhibits EP300, an acetyltransferase involved in both post-translational and epigenetic regulation of autophagy [27, 37]. Our data also confirmed that trehalose increases nuclear levels of TFEB, whereas spermidine reduces protein acetylation. Interestingly, TRE and SP are considered caloric restriction mimetics, since they exert similar functions and modulate similar molecular targets of caloric restriction [26, 33]. It was previously demonstrated that exercise and caloric restriction exert cardioprotective effects against chemotherapy-induced cardiotoxicity [3, 46], further supporting the observed beneficial effects of TRE and SP.

Interestingly, we found that TRE treatment significantly attenuates DOX-induced mitochondrial damage and significantly stimulates mitophagy, a specific form of autophagy that removes mitochondria [10]. Mitophagy is an essential mechanism for the removal of damaged mitochondria and their turnover. Mitophagy activation was previously shown to limit I/R injury, heart failure, diabetic cardiomyopathy and vascular disorders [1, 10, 41]. We propose that autophagic flux inhibition by DOX contributes in the development of mitochondrial damage and dysfunction also by impairing mitophagy, which is fundamental for mitochondrial repair following fission of damaged portions. It is also possible that DOX causes mitochondrial damage, which cannot be repaired because of insufficient autophagic response and flux activation. TRE and SP may boost autophagic response and flux thereby reducing myocardial injury. TRE and SP may also directly modulate mitophagy machinery stimulating this process, independently of flux reactivation. Future studies are needed to better clarify these aspects. In this regard, our study is limited to a six-week observation period, following doxorubicin administration, which was previously found to be appropriate to evaluate the chronic cardiotoxic effects of DOX. Future studies with extended follow-up observations will be required to determine whether sustained autophagy activation continues to confer cardioprotection in the long-term.

Our data suggest that mitochondrial biogenesis is triggered by DOX treatment, likely because mitophagy is insufficient for mitochondrial quality control. However, biogenesis activation appears to be inadequate for preserving mitochondrial function in any case. In contrast, biogenesis seems to be less activated in the heart of mice treated with DOX and TRE, likely because mitophagy activation promotes mitochondrial repair and preserves mitochondrial function. Further studies are also needed to investigate this issue.

In the evolving landscape of cardioprotective strategies against anthracycline-induced cardiotoxicity, TRE and SP emerge as innovative and promising approaches. These naturally occurring compounds are already FDA-approved as dietary ingredients, and their safety has been evaluated in human studies both via oral and intravenous administration. Previous clinical studies already demonstrated both safety and efficacy of these compounds against neurological and vascular diseases [17, 21]. Importantly, the doses of trehalose and spermidine used in this study are within the range of previously reported cardioprotective regimens in preclinical models and potentially in line with those tested in patients. Although direct pharmacokinetic comparisons between murine and human dosing require careful allometric scaling, the regimens employed here are consistent with those shown to be safe in humans [17, 18, 28, 33]. On the other hand, Tat-Beclin 1 D11 currently represents an experimental autophagy-inducing peptide never tested in the clinical setting. In our study, this compound was primarily used as a mechanistic tool to selectively induce autophagy by directly interfering with autophagy machinery, and, therefore, further support the importance of autophagy activation for the prevention of DOX-induced cardiotoxicity. While Tat-Beclin 1 D11 strengthens causal inference regarding autophagic flux restoration, its translational development will require dedicated pharmacokinetic, safety, and delivery studies.

Our study suggests that TRE or SP may represent promising candidates for the prevention of anthracycline-induced cardiotoxicity in patients with cancer, offering a novel pathway to cardiac protection without the established risks associated with other agents, such as dexrazoxane. Despite the fact that dexrazoxane is recognized to exert cardioprotective effects, as recently confirmed by a study showing an association of this drug with a cardioprotective effect after nearly 20 years since initial anthracycline exposure [4], and it is the only FDA-approved cardioprotection for patients undergoing anthracycline therapy, its application, especially in pediatric populations, still requires additional investigations, as suggested by a recent meta-analysis [5].

Interestingly, we also evaluated the effects of our autophagy activators in a preclinical cancer model that more closely reflects the complexity of cancer-bearing subjects [14, 16]. We demonstrated that TRE, SP, and TAT do not compromise the antineoplastic efficacy of doxorubicin (DOX), while effectively mitigating its cardiotoxic effects, highlighting their potential as safe adjuncts in cancer therapy. Since TRE or SP are FDA-approved for over-the-counter use, their application as dietary supplements for oncology patients might potentially offer a safe and accessible cardioprotective strategy. We strongly advocate for future clinical trials to further investigate this hypothesis, aiming to apply these findings directly to patient care, after a rigorous evaluation of their safety and efficacy.

## Supplementary Information

Below is the link to the electronic supplementary material.

Supplementary file1 (DOCX 32299 kb)

## Data Availability

The data supporting this article will be made available upon reasonable request to the corresponding author.

## Abbreviations

CQ: Chloroquine
DOX: Doxorubicin
FS: Fractional shortening
LC3: Microtubule-associated proteins 1A/1B light chain 3B
p62: Sequestosome 1
PGC1α: Peroxisome proliferator-activated receptor gamma coactivator 1-alpha
SP: Spermidine
TAT: Tat-Beclin 1 D11
TRE: Trehalose
