# Supplementary material for: Pharmacological reactivation of autophagic flux by natural compounds or synthetic cell-permeable peptide prevents doxorubicin-induced cardiomyopathy
Source: Basic Res Cardiol. 2026 Mar 29;121(3):523–39. doi: 10.1007/s00395-026-01174-9 (PMC13186803; doi:10.1007/s00395-026-01174-9)
Supplement: Supplementary file 1 — Supplementary file1 (DOCX 32299 kb) [file 395_2026_1174_MOESM1_ESM.docx]

**SUPPLEMENTAL MATERIAL**

**Pharmacological reactivation of autophagic flux by natural compounds or synthetic cell-permeable peptide prevents doxorubicin-induced cardiomyopathy**

Schirone et al.

**SUPPLEMENTAL MATERIAL**

**Autophagic Flux Evaluation**

Autophagic flux was assessed by utilizing two methodologies outlined in the "Guidelines for the use and interpretation of assays for monitoring autophagy (4th edition)" [3]. *In vitro,* cardiomyocytes (CMs) were infected for 48 hours with an adenovirus overexpressing mRFP-GFP-LC3, followed by a 24-hour treatment with trehalose at 50 mM, and subsequently, a 24-hour co-treatment with trehalose 50 mM and DOX 15 µM. Cells were then fixed with 4% paraformaldehyde (PFA) at 4°C for 15 minutes and washed with PBS. Imaging in FITC and TRITC channels was performed using a Nikon Eclipse Ni microscope. Yellow and red puncta per cell were manually enumerated in four independent, random microscopic fields for each chamber of a chambered cell culture slide (Falcon). The average number of puncta per cell in each well was considered. In the same experimental condition, we also evaluated autophagic flux in CMs by a western blot analysis for LC3-II, in the presence or absence of bafilomycin (Sigma Aldrich, Milan, Italy), 100 nM for the last 4 hours of DOX treatment.

For the evaluation of autophagic flux in vivo, six weeks following the initial DOX injection, chloroquine (CQ) (Sigma Aldrich) at a dose of 10 mg/kg was administered, four hours before the sacrifice. Proteins were extracted as specified below, and levels of LC3-II were quantified and normalized against the control group (indicated as CTR NT) The autophagic flux was calculated as the ratio of the normalized LC3-II band intensity of the chloroquine-treated group to its corresponding group without chloroquine treatment mean (CTR CQ/CTR NT, DOX CQ/DOX, TRE+DOX CQ/TRE+DOX for trehalose and SP+DOX CQ/SP+DOX for spermidine).

**Cardiomyocytes isolation and culture**

Hearts were extracted from neonatal rats 1-3 days post-delivery, with atria excised and the ventricles finely chopped into small pieces in PBS at room temperature. Subsequently, a commercial kit was utilized to digest the tissue and achieve a single cell suspension (Miltenyi Biotec 130-098-373). Cardiomyocytes were then isolated through negative selection using an antibody-based chromatographic column (Miltenyi Biotec 130-105-420), in accordance with the manufacturer's guidelines. Plates were prepared with a 1% gelatin coating (Sigma G1890), and cardiomyocytes were seeded at a density of 10^6 cells/ml in a specially prepared 'seeding medium' containing 10% horse serum and 100µM BrdU. After 24 hours, once the cells had firmly attached, the medium was replaced with a serum-free 'cardiomyocyte medium' (CM medium). This medium was prepared from freeze-dried DMEM/F-12 (Sigma D0547) supplemented with 0.72 g/l glucose (Sigma G5400), 0.33 g/l sodium pyruvate (Fisher BP356), 0.017 g/l ascorbic acid (Gibco 13080-23), 2 µl of 0.2 M selenite (Sigma S5261), 0.004 g/l transferrin (Sigma T3309), 2 g/l BSA fraction V (Amresco 0332), 3.57 g/l HEPES (Amresco 0511), 2.43 g/l sodium bicarbonate (Sigma S6014), and 10 ml penicillin-streptomycin (Gibco 15070063).

**Electron Microscopy**

Myocardium samples were fixed in 2.5% glutaraldehyde in 0.1M PBS at pH 7.4 for a minimum of 48 hours at 4°C, then rinsed with PBS. For post-fixation, 1.33% osmium tetroxide (Agar Scientific, Stansted, UK) was applied for 2 hours. The samples were then washed twice for 20 minutes to remove any remnants of the osmium tetroxide solution. Dehydration was achieved through a series of ethanol dilutions (30%, 70%, 95%, 3x(100%) v/v). The ethanol was subsequently replaced with propylene oxide (BDH Italia, Milan, Italy). A 50:50 mixture of propylene oxide and epoxy resin Agar 100 (SIC, Rome, Italy) was prepared, and the samples were incubated overnight at 25°C. Next, the samples were embedded in epoxy resin Agar 100 and cured in an oven at 60°C for 48 hours. Epoxy resin blocks were sectioned into semithin slices (1 µm thick), placed on glass slides, and stained with Azur II for preliminary examinations under a light microscope (Carl Zeiss Axioskop‐40, Zeiss, Germany). For transmission electron microscopy (TEM) analysis, ultrathin sections (80–90 nm) were prepared using an ultramicrotome (Leica EM UC6, Vienna, Austria). These sections were collected on 200-mesh copper grids (Assing, Rome, Italy), stained with Uranyless© solution and lead citrate, and imaged with a transmission electron microscope operating at an accelerating voltage of 60 kV (Carl Zeiss EM10, Thornwood, NY), as previously mentioned [1, 2]. Images were captured using a CCD digital camera (AMT CCD, Deben UK Ltd, Suffolk, UK). The number of damaged mitochondria was evaluated by the presence of at least one morphological alteration. The latter includes mild (clearing matrices, small stack of abnormally osmiophilic closely apposed cristae) and severe alteration/degeneration (large stack of abnormally osmiophilic closely apposed cristae, loss of cristae, large vacuoles, notorious myelin figures). Mitophagic bodies were also quantified in each microscopic field by image J. Mitochondrial analysis was performed on 13 microscopic fields (original magnification 10900X) for each sample.

**Gravimetric Studies**

The entire hearts were rinsed in PBS to eliminate any blood, then dried and weighed. Subsequently, the tibial length was measured to normalize the heart weight to the actual size of the mouse.

**Masson’s trichrome staining**

A ring-shaped section of the freshly harvested heart was immediately fixed in 4% paraformaldehyde (PFA), embedded in paraffin, and then sectioned into histological slices. The samples were gradually rehydrated and sequentially stained: first with Weigert's iron hematoxylin for 10 minutes, followed by staining in Biebrich scarlet-acid fuchsin solution for 15 minutes, and finally in phosphomolybdic-phosphotungstic acid solution for another 15 minutes. After each staining step, the sections were rinsed in warm water for 15 minutes and then washed in flowing tap water for 10 minutes. Subsequently, the samples were dehydrated in 95% ethanol, cleared in xylene, and mounted with a resinous medium. Imaging was performed using a D-sight microscope (Menarini) in bright field mode. Fibrosis was quantified using ImageJ software by performing color deconvolution to measure the ratio between the blue and red-colored areas.

**Mitochondrial biogenesis and mitophagy evaluation**

MitoTimer +/- (C57BL/6-Tg(Myh6-DsRed1*/COX8A)40830Rag/J – JAX stock no. 028715) mice were treated with DOX and TRE (as detailed in previous sections) for 3 weeks, after which they were sacrificed for tissue collection. The hearts were immediately embedded in OCT-compound cryogel and frozen. Slices 5 µm thick were prepared, and the samples were fixed with freshly prepared 4% paraformaldehyde (PFA) for 15 minutes at 4°C. Subsequently, the samples were analyzed using fluorescence microscopy (Nikon Eclipse Ni) at wavelengths of 488 nm and 594 nm to enumerate green and red dots, respectively. Green dots indicate mitochondria produced recently (< 48 hours), while red dots represent older mitochondria (> 48 hours). The ratio of green- to red-only dots was calculated to evaluate mitochondrial biogenesis.

To assess mitophagy in a living context, we prepared 5 µm thick sections, which were subsequently fixed with 4% paraformaldehyde (PFA). These sections were then incubated in a blocking solution for one hour. Following this, we applied anti-LC3 antibody (Novus Biologicals 100-2220) and allowed it to incubate overnight, as previously reported [5]. The Alexa Fluor™ 647 secondary antibody (Thermo Fisher Scientific) was then added and left to incubate for one hour. After several washing steps, the samples were mounted. Image acquisition of MitoTimer’s spontaneous 594 nm signal and LC3’s 647 nm was performed using the Zeiss Confocal software (Zen 3.0 Blue edition). The images presented are shown in false colours for clarity, as detailed in the figure legend.

**Real-time PCR quantification of mitochondrial DNA**

Total DNA was extracted from mouse hearts using a specific commercially available kit (Promega, A1125) following the manufacturer’s instructions. The mtDNA content was quantified by real-time PCR for cytochrome b using the ViiA 7 Real-Time PCR System (Applied Biosystem, Foster City, CA, USA) and SYBR Select Master Mix (Applied Biosystem). Primer sequences used are: 5’-CCACTTCATCTTACCATTTATTATCGC-3’ (forward primer) and 5’-TTTTATCTGCATCTGAGTTTAA-3’ (reverse primer) for cytochrome b, and 5’-CTGCCTGACGGCCAGG-3’ (forward primer) and 5’- CTATGGCCTCAGGAGTTTTGTC-3’ (reverse primer) for genomic β-actin.

**TUNEL assay**

Paraffin-embedded heart sections underwent a meticulous preparation process beginning with the removal of paraffin. This was achieved by heating the slides in an oven at 37°C for 20 minutes, followed by two xylene washes, each lasting 5 minutes. Subsequently, the sections were rehydrated through a series of ethanol washes at decreasing concentrations (100%, 95%, 85%, 70%, 50%), each for 3 minutes, then briefly immersed in 0.85% NaCl and PBS for 5 minutes each.

Samples were fixed in 4% formaldehyde in PBS for 15 minutes to preserve cellular structures. This was followed by two PBS washes to remove any residual fixative. The sections were then permeabilized with 0.2% Triton X-100 in PBS for 10 minutes and washed again with PBS three times to remove any excess permeabilization agent. The sections were blocked with 4% BSA in PBS for 30 minutes to prevent nonspecific binding of the antibodies. They were then incubated with a primary anti-Troponin T antibody (Invitrogen PA5-102358) diluted in 1% BSA in PBS overnight at +4°C, ensuring specific binding to the target antigen. Following primary antibody incubation, the sections were treated with a suitable secondary antibody, either the Alexa Fluor 594 conjugated goat anti-rabbit IgG (H+L) Highly Cross-Adsorbed (A-11037) or the Alexa Fluor 488 conjugated goat anti-mouse IgG (H+L) Highly Cross-Adsorbed (A-11029), depending on the primary antibody used. This was to visualize the antigen-antibody complexes.

A TUNEL assay was then performed using the Dead END Fluorometric TUNEL System (G3250, PROMEGA) according to the manufacturer's instructions. Hoechst 33342 (H3570, Life Technologies) was used for nuclei staining. Finally, the sections were mounted with Vectashield Mounting Medium (H-1000, Vector Laboratories) to preserve fluorescence and prevent photobleaching. Imaging was conducted on a Nikon Eclipse Ni fluorescence microscope at wavelengths of 359 nm, 488 nm, and 594 nm.

**Western Blot**

The cell culture medium was discarded, and the culture plates were rinsed with PBS. Cells were then harvested in ice-cold RIPA lysis buffer (comprising 50 mM TRIS at pH 7.4, 150 mM NaCl, 1 mM EDTA, 1% Triton X-100, and 10% glycerol in deionized water) enriched with phosphatase (Sigma 04906845001) and protease inhibitors (Sigma 11873580001). In a similar fashion, whole hearts were pulverized in 200 µl of RIPA lysis buffer using a mortar. The resultant homogenates were agitated for 1 hour at 4°C and subsequently clarified by centrifugation at 14,460 g for 25 minutes. The supernatants were collected, combined with 4x Laemmli buffer (BioRad 1610747) and 2.5% β-mercaptoethanol, and boiled for 8 minutes. Protein concentrations were determined using the Bradford colorimetric assay (BioRad 5000001), followed by separation on 6 to 15% SDS-PAGE and transfer onto polyvinylidene difluoride membranes. Nuclear and cytoplasmic proteins from hearts were separated as described above [4] and normalized for Histone 3 and GAPDH, respectively. The membranes were blocked with 5% non-fat dry milk in 0.05% Tween 20-containing TBS for 90 minutes, then incubated overnight with the designated primary antibodies. HRP-conjugated mouse and rabbit secondary antibodies were diluted 1:2000 in the blocking solution and applied to the membranes for 90 minutes. Membranes were visualized using the enhanced chemiluminescence kit (Amersham ECL Select™ RPN2235). Image acquisition and densitometry analysis were conducted using ChemiDoc and Image J software. The following primary antibodies were used: cl-CAS3 (CS-9661), LC3 (M186-3), p62 (A-H00008878-M01), PGC-1α (I-PA5-38021), Vinculin (sc-25336), LAMP-2 (I-PA1-655), TFEB (B, A303-673A), GAPDH (sc-32233), H3 (CS-9715S), Acetylated lysine (CS-9441) [A = Abnova; B = Bethyl; CS = Cell Signaling; I = Invitrogen; M = MBL (Japan); NB = Novus Biologicals; SC = Santa Cruz]. Anti-mouse (CS-7076) and anti-rabbit (CS-7074) HRP-linked antibodies were purchased from Cell Signaling Technology.

**SUPPLEMENTAL FIGURES LEGENDS**

**
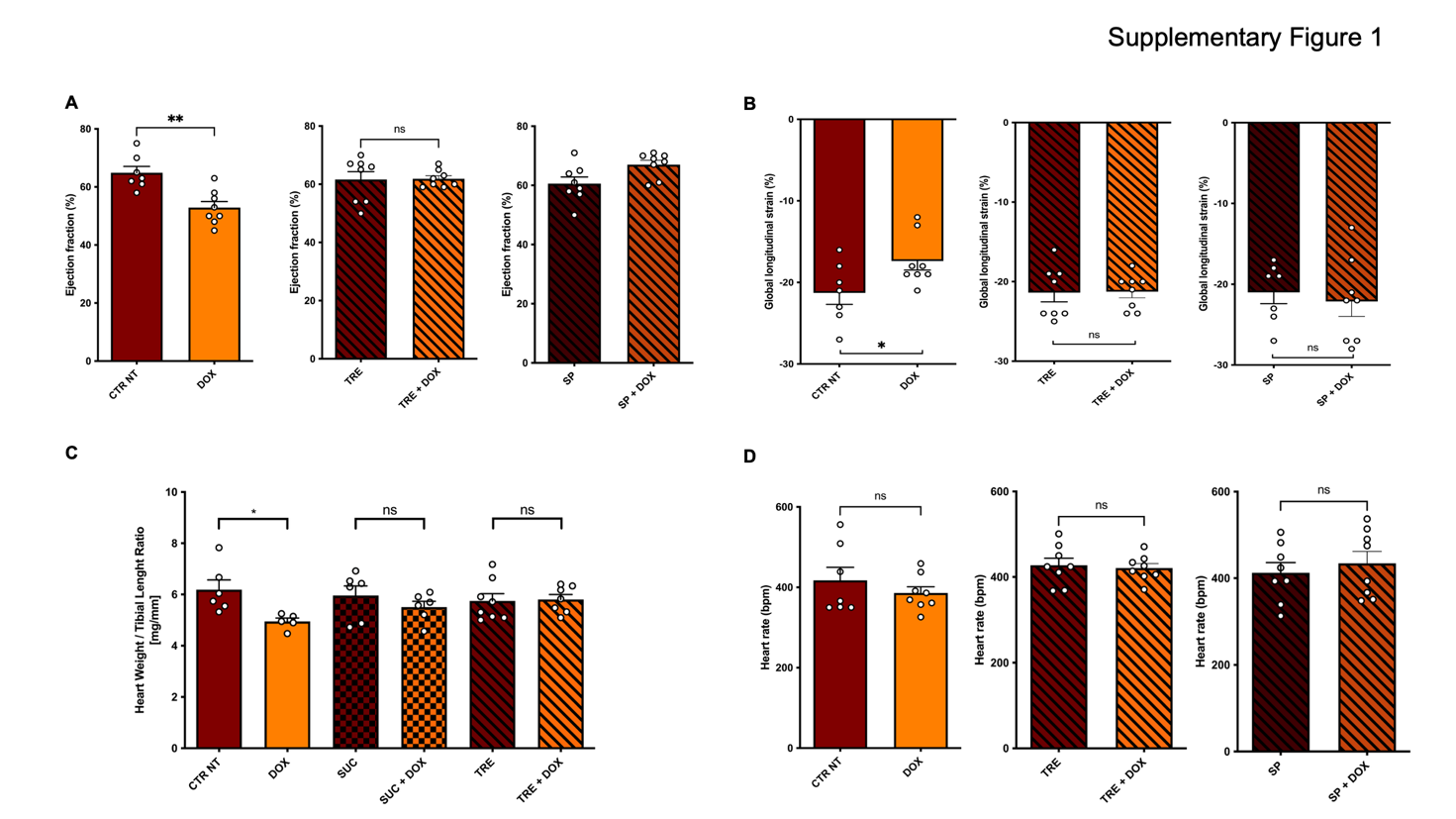
Supplementary Figure 1**

(**A**) Ejection fraction and (**B**) global longitudinal strain (GLS) from mice receiving 6 weeks of DOX, with or without trehalose (TRE) or spermidine (SP) (N=7-8). (**C**) Heart weights from mice receiving 6 weeks of DOX with or without trehalose (TRE) or sucrose (SUC) treatment. Heart weight was normalized on tibial length to account for body size (N= 5-8). (**D**) Heart rate from mice receiving 6 weeks DOX, with or without trehalose (TRE) or spermidine (SP) (N=7-8). Data represent mean ± SEM. Data were analyzed with Student’s T-test. *P ≤ 0.05; **P≤ 0.01; ns= non-significant (P > 0.05). Legend: CTR NT = Control mice not-treated; DOX = Control mice treated with DOX; TRE = mice treated with trehalose; SP = mice treated with spermidine; SP+DOX = mice treated with DOX and spermidine; TRE+DOX = mice treated with DOX and trehalose; SUC = mice treated with sucrose; SUC+DOX = mice treated with sucrose and DOX.

**
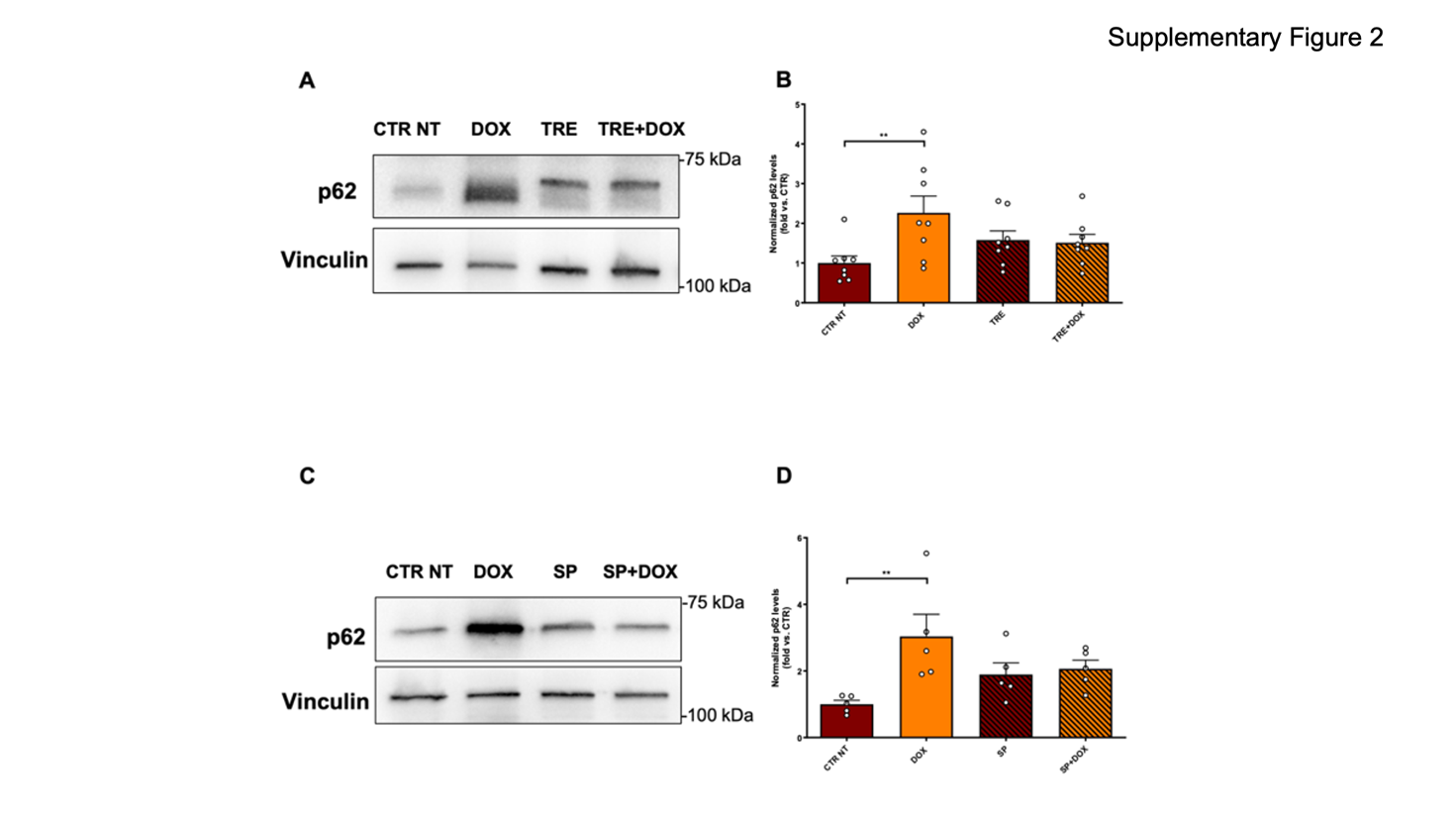
Supplementary Figure 2**

(**A-D**) Representative western blot for p62 autophagy marker in myocardial lysates (**A, C**) and corresponding quantification in mice treated with trehalose (**A-B**) or spermidine (**C-D**). Data represent mean ± SEM; (N = 8) (**A-B**); (N = 5) (**C-D**). Data were analyzed with one-way ANOVA with a Bonferroni post-hoc test. **P ≤ 0.01.

Legend: CTR NT = Control mice not-treated; DOX = Control mice treated with DOX; TRE = mice treated with trehalose; TRE+DOX = mice treated with DOX and trehalose; SP = mice treated with spermidine; SP+DOX = mice treated with spermidine and DOX.

**
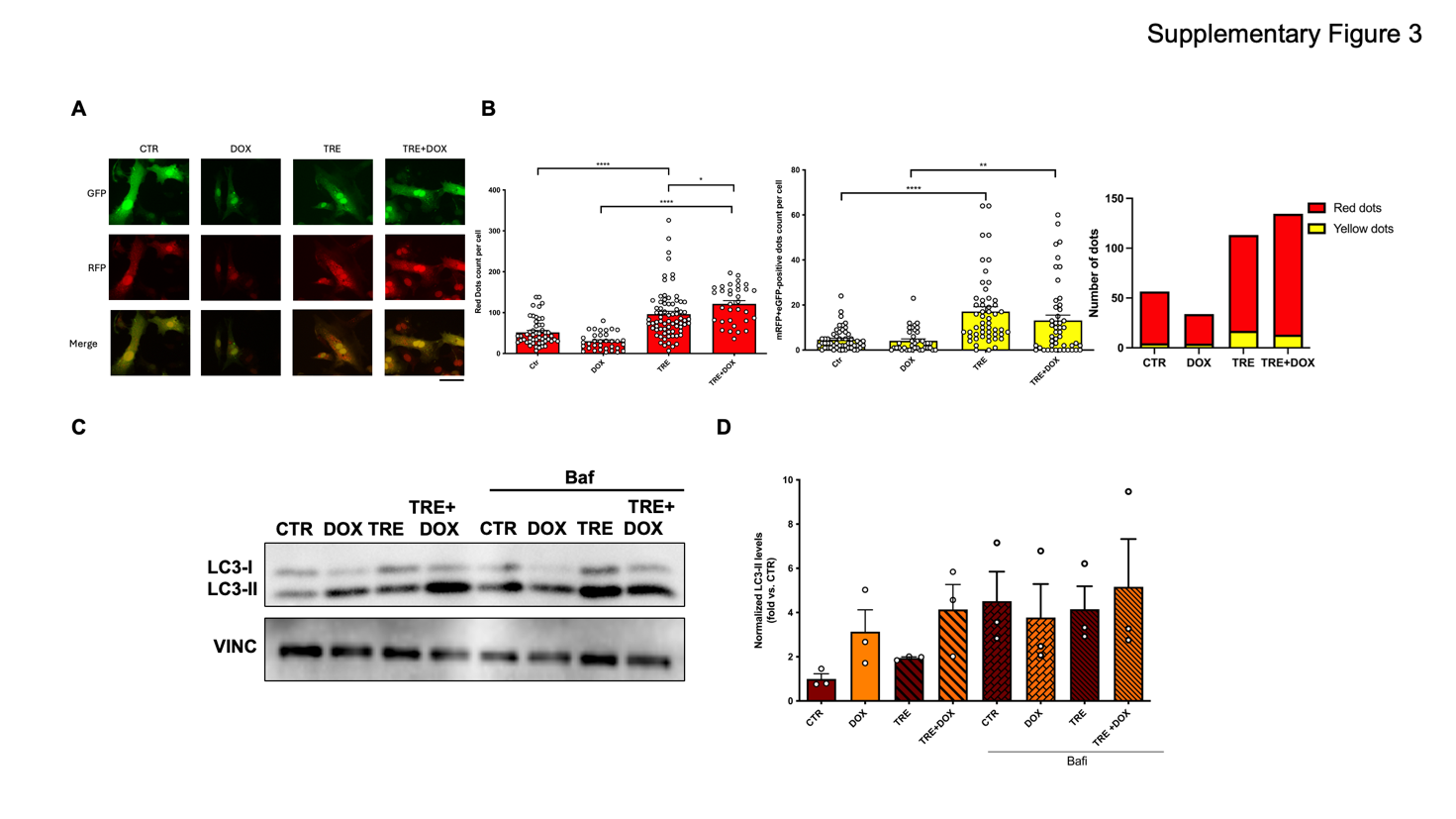
Supplementary Figure 3**

**(A-B)** Neonatal rat primary cardiomyocytes (CMs) were infected with ad-mRFP-eGFP-LC3b for 48 hours, then treated with trehalose 50 mM for 24h, then 4 hours with DOX 15 µM + trehalose 50 mM. Random cells’ images were captured with fluorescence microscopy, and the numbers of red and yellow (i.e. red + green) dots were counted. Data represent mean dots number per cell ± SEM (N = 33-73 cells from 4 independent samples). Scalebar = 50 µm. (**C-D**). Representative western blot for LC3 and corresponding quantification. Bafilomycin (Baf) was added at 100 nM for the last 4 hours of treatment (N=3). Data were analyzed with one-way ANOVA with a Bonferroni post-hoc test. *P ≤ 0.05; **P ≤ 0.01; ****P ≤ 0.0001. Legend: CTR= Control cells not-treated; DOX = Control cells treated with DOX; TRE = cells treated with trehalose; TRE+DOX = cells treated with DOX and trehalose.

**
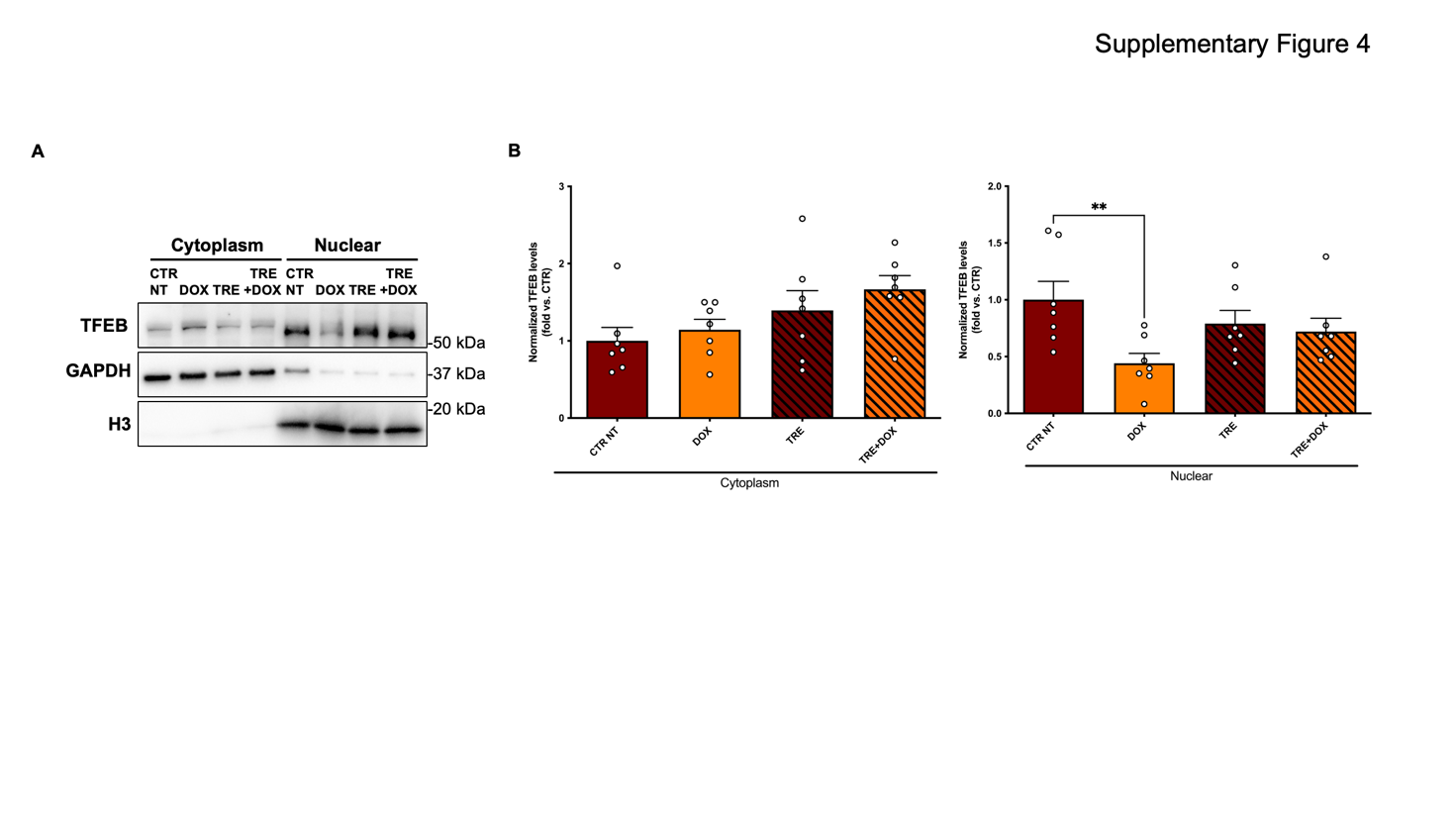
Supplementary Figure 4**

(**A**) Representative western blot for TFEB in cytoplasmic and nuclear myocardial lysates of mice treated with Doxorubicin (DOX), in the presence or absence of trehalose (TRE), and corresponding quantification (**B**). Data represent mean ± SEM (N = 7); Data were analyzed with one-way ANOVA with a Bonferroni post-hoc test. **P ≤ 0.01. Legend: CTR NT = Control mice not treated; DOX = Control mice treated with DOX; TRE = mice treated with trehalose; TRE+DOX = mice treated with DOX and trehalose.

**
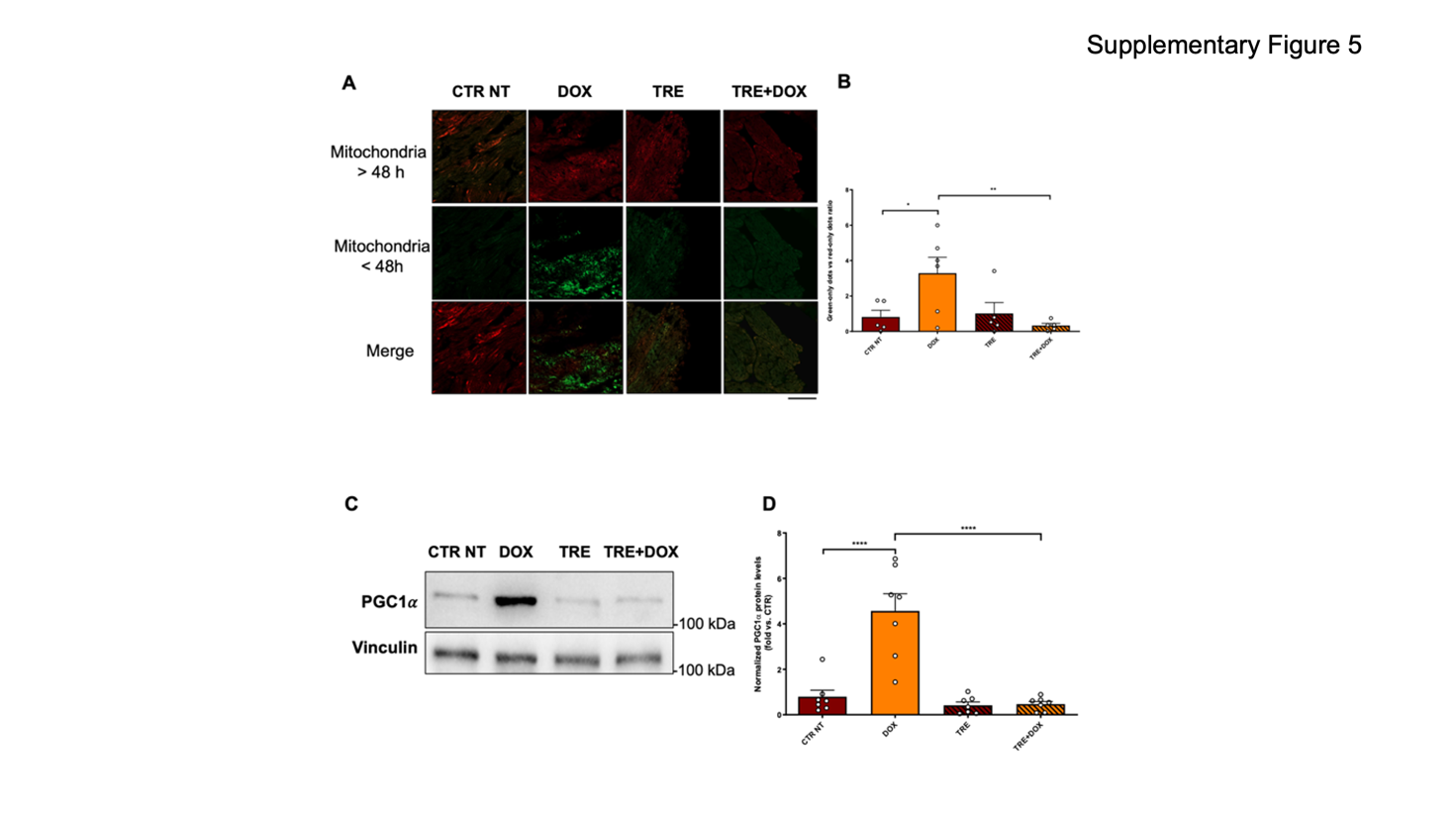
Supplementary Figure 5**

**(A-B)** Mitochondrial biogenesis evaluation by fluorescence analyses of red/green dots in MitoTimer mice treated with doxorubicin (DOX) and trehalose. Data represent mean ± SEM (N= 5-6). Scalebar = 70µm; **(C-D)** Representative western blot for PGC1-α from myocardial lysates and corresponding quantification. Data represent mean ± SEM (N = 7); Data were analyzed with one-way ANOVA with a Bonferroni post-hoc test. *P ≤ 0.05; **P ≤ 0.01 ****P ≤ 0.0001. Legend: CTR NT = Control mice not-treated; DOX = Control mice treated with DOX; TRE = mice treated with trehalose; TRE+DOX = mice treated with DOX and trehalose.

**
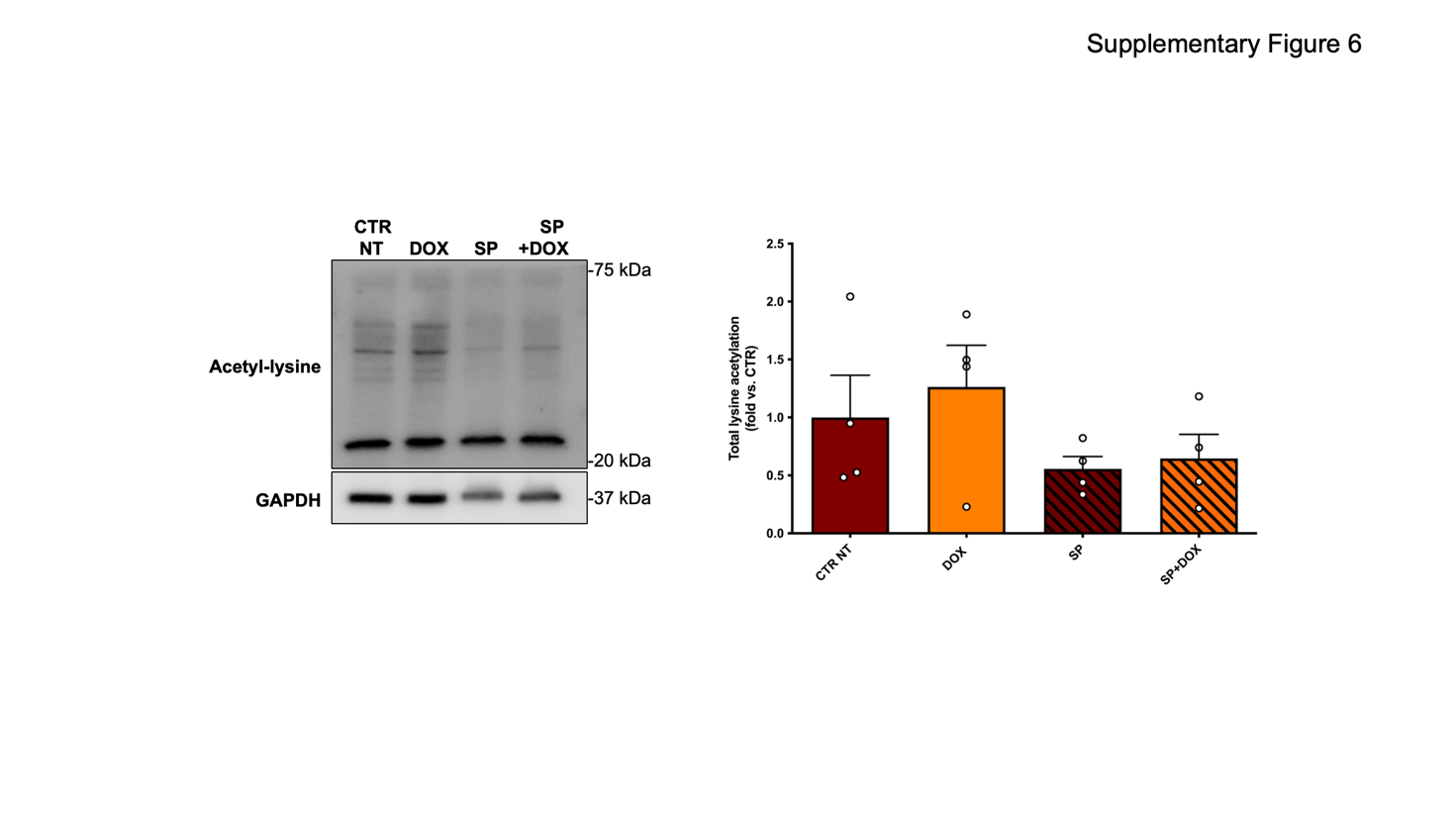
**

**Supplementary Figure 6**

Representative western blot for total lysine acetylation and corresponding quantification. Data represent mean ± SEM (N = 4). Legend: CTR NT = Control mice not-treated; DOX = Control mice treated with DOX; SP = mice treated with spermidine; SP+DOX = mice treated with DOX and spermidine.

**
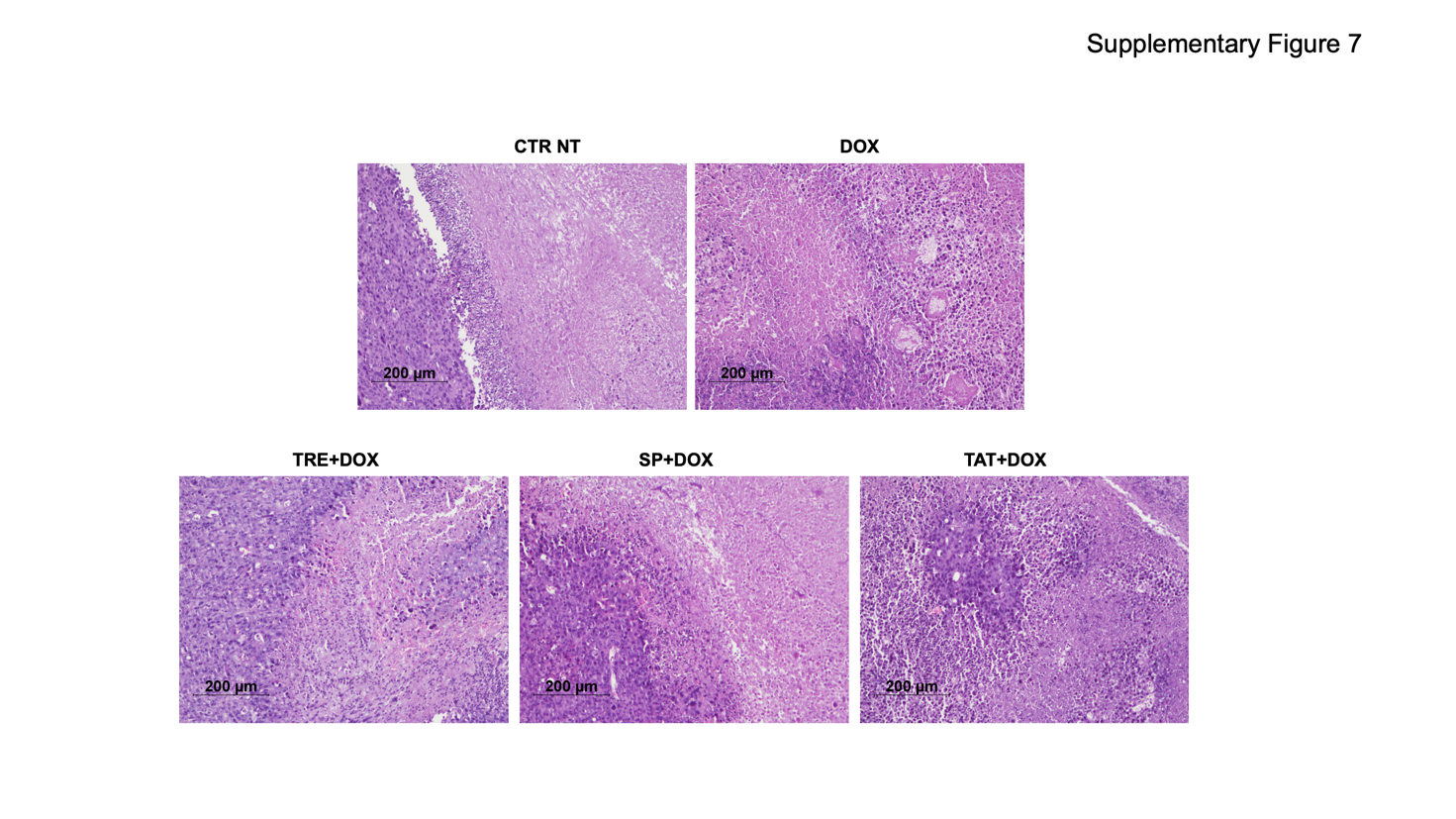
Supplementary Figure 7**

Representative hematoxylin and eosin (H&E) images of excised tumors collected from C57BL/6N mice of the following groups: control non-treated (CTR NT), doxorubicin (DOX), trehalose (TRE) + DOX, Spermidine (SP) + DOX, and Tat-Beclin 1 D11 (TAT) + DOX. Scale bar = 200 µm.

**
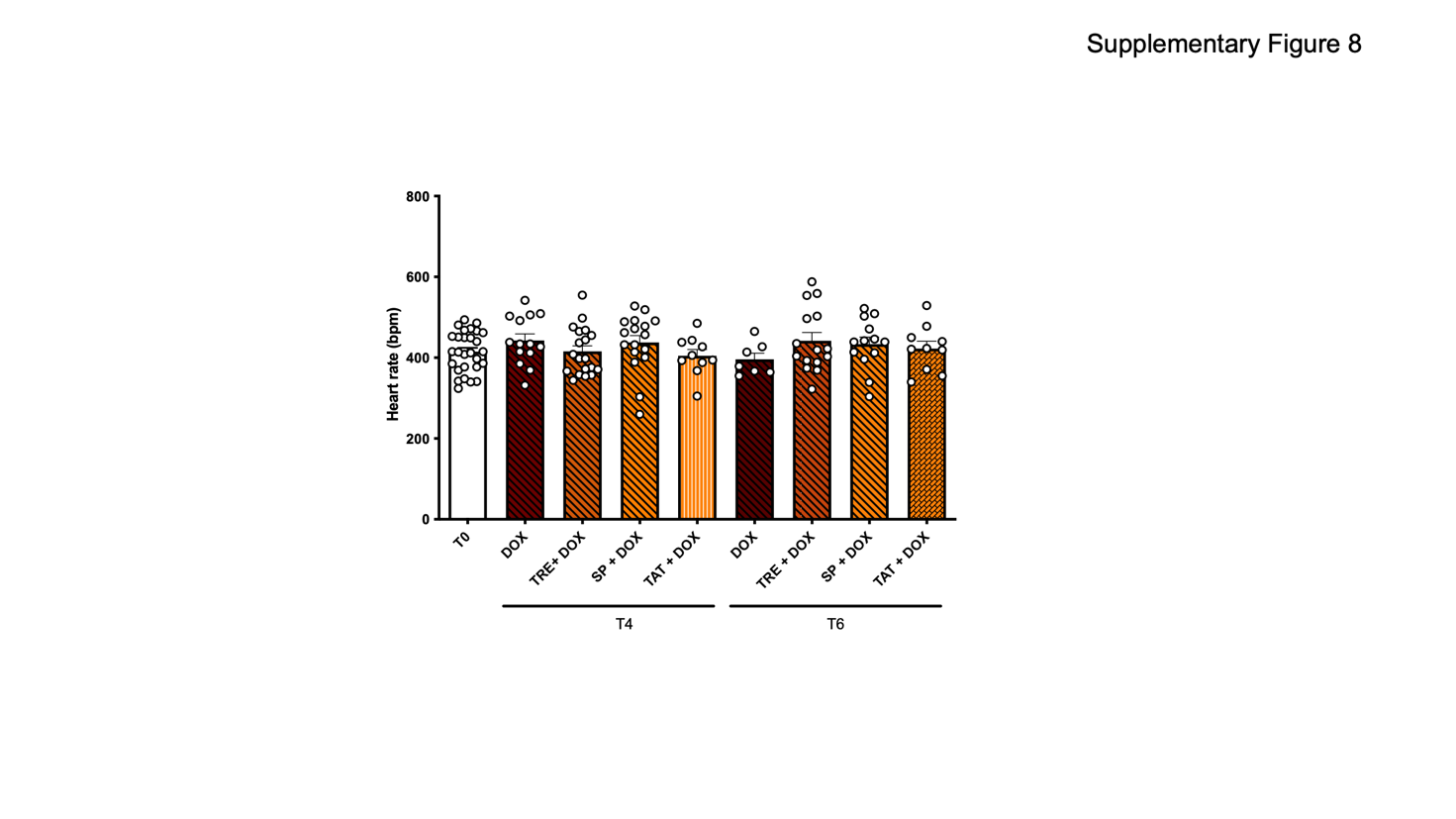
Supplementary Figure 8**

Heart rate at baseline (T0) and at 4 (T4) and 6 (T6) weeks after doxorubicin (DOX) treatment in C57BL/6N mice with subcutaneous injection of E0771 breast cancer cells (N = 28 at T0; T4: DOX N = 14, TRE+DOX N = 19, SP+DOX N = 17, TAT+DOX N = 10; T6: DOX N = 7, TRE+DOX N = 15, SP+DOX N = 13, TAT+DOX N = 10). Legend: DOX, doxorubicin; DOX + trehalose (TRE), DOX + spermidine (SP) and DOX + Tat-Beclin 1 D11 (TAT).

**
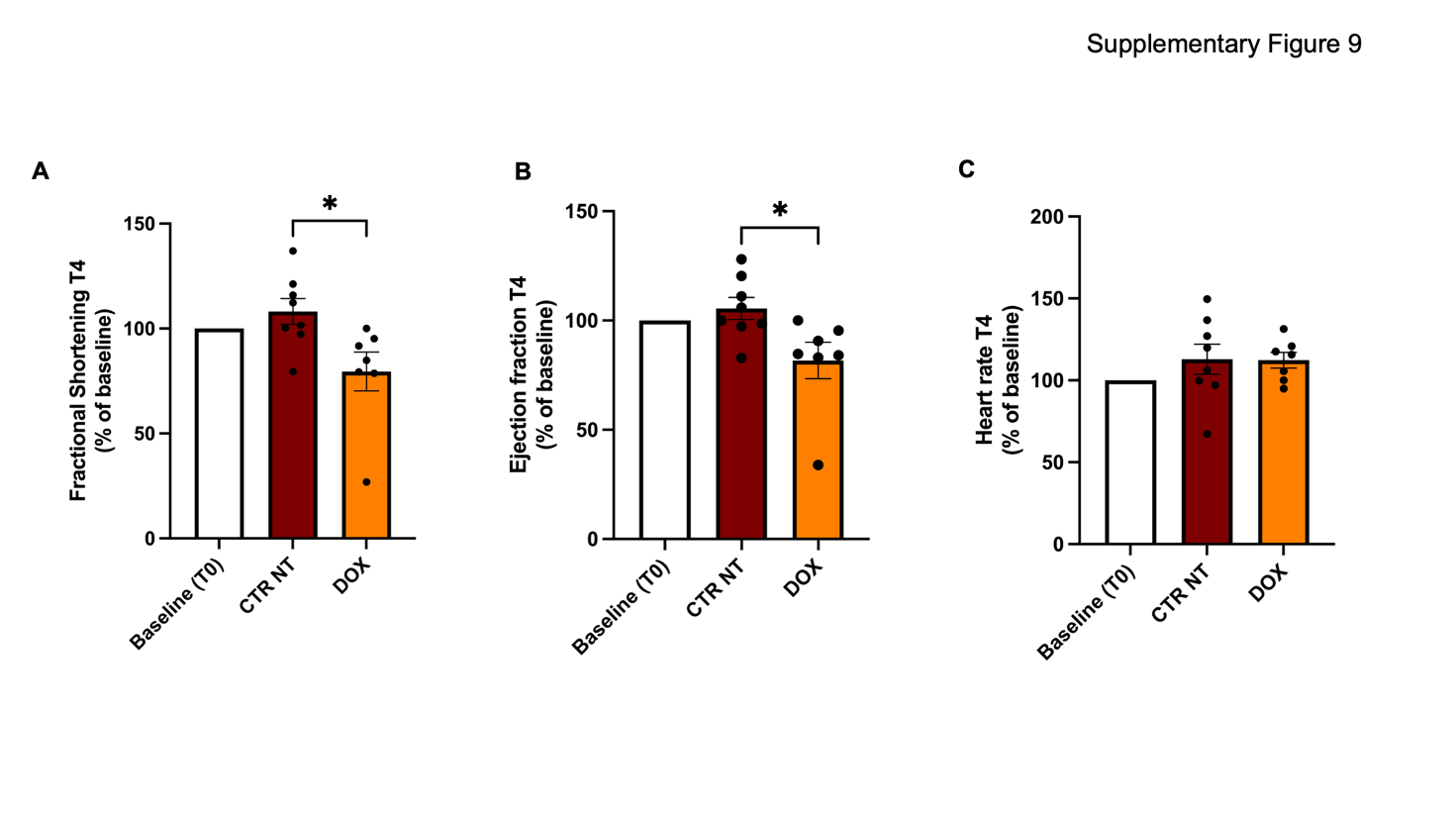
**

**Supplementary Figure 9**

Percentage changes in fractional shortening (**A**), ejection fraction (**B**) and heart rate (**C**) measured at T4 (4 weeks) compared to T0 (baseline, 0 weeks) after the first doxorubicin injection in a syngeneic model of subcutaneous injection of EO771 breast cancer cells in C57BL/6N mice. Data are shown for untreated controls (CTR NT) and for animals treated with doxorubicin (DOX). Values represent relative variations from baseline (T0). Data represent mean ± SEM. (CTR NT N=8; DOX N=7); *P ≤ 0.05 obtained by Student’s T-test.

**Supplementary Tables**

**Supplementary Table 1 – Animal groups - trehalose experiments Figure 1**

|  | Number | Males | Age [Mean + SEM] |
| --- | --- | --- | --- |
| Control untreated | 14 | 50% | 2.5 ± 0.02 |
| Doxorubicin | 16 | 31% | 2.2 ± 0.03 |
| Sucrose | 7 | 43% | 2.6 ± 0.09 |
| Sucrose + Doxorubicin | 8 | 50% | 2.2 ± 0.09 |
| Trehalose | 13 | 46% | 2.6 ± 0.05 |
| Trehalose +  Doxorubicin | 15 | 40% | 2.1 ± 0.05 |

**Supplementary Table 2 – Animal groups – spermidine experiments Figure 4**

|  | Number | Males | Age [Mean + SEM]] |
| --- | --- | --- | --- |
| Control untreated | 11 | 36% | 3 ± 0.12 |
| Doxorubicin | 14 | 43% | 3 ± 0.1 |
| Spermidine | 11 | 36% | 3 ± 0.12 |
| Spermidine + Doxorubicin | 10 | 40% | 3.1 ± 0.13 |

**Supplementary Table 3 – Animal groups - Tat-Beclin 1 D11 experiments Figure 5**

|  | Number | Males | Age [Mean + SEM]] |
| --- | --- | --- | --- |
| Control untreated | 15 | 47% | 2.7 ± 0.12 |
| Doxorubicn | 17 | 47% | 2.8 ± 0.28 |
| Tat-Beclin 1 D11 | 8 | 50% | 3 ± 0.12 |
| Tat-Beclin 1 D11 + Doxorubicin | 10 | 50% | 2.6 ± 0.12 |

**REFERENCES**

1. Cottignoli V, Relucenti M, Agrosì G, Cavarretta E, Familiari G, Salvador L, Maras A (2015) Biological Niches within Human Calcified Aortic Valves: Towards Understanding of the Pathological Biomineralization Process. Biomed Res Int 2015:542687 doi:10.1155/2015/542687

2. Grimaldi A, Serpe C, Chece G, Nigro V, Sarra A, Ruzicka B, Relucenti M, Familiari G, Ruocco G, Pascucci GR, Guerrieri F, Limatola C, Catalano M (2019) Microglia-Derived Microvesicles Affect Microglia Phenotype in Glioma. Front Cell Neurosci 13:41 doi:10.3389/fncel.2019.00041

3. Klionsky DJ, Abdel-Aziz AK, Abdelfatah S, Abdellatif M, Abdoli A, Abel S, Abeliovich H, Abildgaard MH, Abudu YP, Acevedo-Arozena A, Adamopoulos IE, Adeli K, Adolph TE, Adornetto A, Aflaki E, Agam G, Agarwal A, Aggarwal BB, Agnello M, Agostinis P, Agrewala JN, Agrotis A, Aguilar PV, Ahmad ST, Ahmed ZM, Ahumada-Castro U, Aits S, Aizawa S, Akkoc Y, Akoumianaki T, Akpinar HA, Al-Abd AM, Al-Akra L, Al-Gharaibeh A, Alaoui-Jamali MA, Alberti S, Alcocer-Gómez E, Alessandri C, Ali M, Alim Al-Bari MA, Aliwaini S, Alizadeh J, Almacellas E, Almasan A, Alonso A, Alonso GD, Altan-Bonnet N, Altieri DC, Álvarez É, Alves S, Alves da Costa C, Alzaharna MM, Amadio M, Amantini C, Amaral C, Ambrosio S, Amer AO, Ammanathan V, An Z, Andersen SU, Andrabi SA, Andrade-Silva M, Andres AM, Angelini S, Ann D, Anozie UC, Ansari MY, Antas P, Antebi A, Antón Z, Anwar T, Apetoh L, Apostolova N, Araki T, Araki Y, Arasaki K, Araújo WL, Araya J, Arden C, Arévalo MA, Arguelles S, Arias E, Arikkath J, Arimoto H, Ariosa AR, Armstrong-James D, Arnauné-Pelloquin L, Aroca A, Arroyo DS, Arsov I, Artero R, Asaro DML, Aschner M, Ashrafizadeh M, Ashur-Fabian O, Atanasov AG, Au AK, Auberger P, Auner HW, Aurelian L, et al. (2021) Guidelines for the use and interpretation of assays for monitoring autophagy (4th edition). Autophagy 17:1-382 doi:10.1080/15548627.2020.1797280

4. Schirone L, Vecchio D, Valenti V, Forte M, Relucenti M, Angelini A, Zaglia T, Schiavon S, D'Ambrosio L, Sarto G, Stanzione R, Mangione E, Miglietta S, Di Bona A, Fedrigo M, Ghigo A, Versaci F, Petrozza V, Marchitti S, Rubattu S, Volpe M, Sadoshima J, Frati L, Frati G, Sciarretta S (2023) MST1 mediates doxorubicin-induced cardiomyopathy by SIRT3 downregulation. Cell Mol Life Sci 80:245 doi:10.1007/s00018-023-04877-7

5. Stotland A, Gottlieb RA (2016) alpha-MHC MitoTimer mouse: In vivo mitochondrial turnover model reveals remarkable mitochondrial heterogeneity in the heart. J Mol Cell Cardiol 90:53-58 doi:10.1016/j.yjmcc.2015.11.032
